# *KCNH3* Loss-of-Function Variant Associated with Epilepsy and Neurodevelopmental Delay Enhances Kv12.2 Channel Inactivation

**DOI:** 10.3390/ijms26104631

**Published:** 2025-05-13

**Authors:** Christiane K. Bauer, Arne Bilet, Frederike L. Harms, Robert Bähring

**Affiliations:** 1 Institute of Cellular and Integrative Physiology, University Medical Center Hamburg-Eppendorf, 20246 Hamburg, Germany; 2 Institute of Human Genetics, University Medical Center Hamburg-Eppendorf, 20246 Hamburg, Germany

**Keywords:** HELK2, EAG, C-type inactivation, KCNH2, HERG, Kv11.1 A561V, ICA-105574

## Abstract

A de novo missense variant in *KCNH3* has been identified in a patient with neurological symptoms including seizures. Here, we confirm the previously reported loss-of-function features for the associated Kv12.2 mutant A371V and investigate the underlying mechanism. Loss of function was not rescued by low temperature during channel biogenesis. Elevated external K^+^ reduced the rectification of Kv12.2 conductance as predicted by the GHK current equation, allowing the detection of currents mediated by homomeric A371V Kv12.2 channels and a detailed biophysical analysis of the mutant. Compared to wild-type, the voltage dependences of activation and deactivation of A371V Kv12.2 channels were shifted in the positive direction by 15 to 20 mV. Moreover, A371V Kv12.2 channels exhibited accelerated inactivation kinetics combined with a dramatic negative shift in the voltage dependence of inactivation by more than 100 mV. Even in heteromeric wild-type + A371V Kv12.2 channels, inactivation was enhanced, leading to a significant current reduction at physiological potentials. Our Kv12.2 data show similarities to Kv11 channels regarding C-type inactivation and differences regarding the sensitivity to external K^+^ and pharmacological inhibition of inactivation. The gating modification caused by the A371V amino acid substitution in Kv12.2 renders loss of function voltage-dependent, with a possible impact on neuronal excitability and firing behavior.

## 1. Introduction

Recently, a de novo missense variant in the *KCNH3* gene was identified in a young female patient presenting with developmental delay, autistic behavior, nocturnal seizures, and insomnia [1]. It was the first clear link between *KCNH3* and a human disease. *KCNH3* encodes the voltage-gated potassium channel Kv12.2, and the functional characterization of the variant-related p.Ala371Val (A371V) mutant suggested a complete loss of function (LoF) and suppression of Kv12.2 currents in a dominant negative manner [1]. The neurological symptoms of the patient correlated with the predominant expression of *KCNH3* in the human brain [2,3] and with the observation of epileptic activity in *Kcnh3*^−/−^ and *Kcnh3*^+/−^ mice [4]. Moreover, the observed insomnia phenotype of the patient [1] may be related to the disturbance of the characteristic day–night rhythm of neuronal activity observed in circadian pacemaker neurons of *Kcnh3* knockout mice [5]. Of note, heterozygous LoF variants in the related *KCNH2* gene (HERG), originally identified for their involvement in cardiac arrhythmia [6,7], may also be related to epilepsy [8,9,10], including one variant which causes an A561V amino acid substitution in Kv11.1 [11,12], homologous to the recently reported A371V amino acid substitution in Kv12.2. The relevant alanine residue is conserved within the ether-à-go-go (EAG) subfamily, a distinct group of voltage-gated potassium (Kv) channels, comprising Kv10 (*KCNH1*, eag), Kv11 (*KCNH2*, erg), and Kv12 (*KCNH3*, elk) subfamily members (Supplementary Figure S1A; [13,14]).

As with all Kv channels, EAG channel α-subunits possess six transmembrane segments (S1–S6) with cytoplasmic N- and C-termini (Supplementary Figure S1B). The EAG family-specific domains are an N-terminal Per-Arnt-Sim (PAS) domain, which, together with a preceding Cap domain, is often referred to as the EAG domain, and a C-terminal cyclic nucleotide binding homology domain (cNBHD) [14,15,16]. All EAG family members possess a turret helix following S5, whereas an extracellular loop extension with three N-glycosylation sites is typical for Kv12.2 ([17]; Supplementary Figure S1). For all Kv channels, four α-subunits must co-assemble to form a functional channel. In the channel tetramer, segments S1–S4 form the voltage-sensing domains, with several positively charged amino acid residues in S4 serving as the primary voltage sensor. The two additional transmembrane segments (S5–S6) along with an intervening pore loop surround the central conduction pathway with a cytoplasmic gate, constituted by the motile distal S6 segments. High conductivity along with high ionic selectivity for K^+^ is mediated by a pore helix and a selectivity filter sequence (GFGN in EAG channels; [13,18]), both located in the pore loop (Supplementary Figure S1). Kv channels are activated (i.e., they transition from a closed to an open state) by membrane depolarization, when voltage sensor motions are directly transmitted to the S6 gate. During prolonged depolarization, Kv channels may undergo inactivation, which is defined as the adoption of a non-conducting state that differs in conformation from the closed state. From this inactivation, the channels can only recover (i.e., become available for activation again) when the membrane is re- or hyperpolarized. Thus, besides the expression level and activation properties, the kinetics and voltage dependence of inactivation critically determine the involvement of a particular Kv channel subtype in cell physiology. Two well-known mechanisms of Kv channel inactivation are (i) the occlusion of the open channel from the cytoplasmic side by an intrinsic N-terminal inactivation domain (N-type inactivation) and (ii) conformational changes of the pore region itself (C-type inactivation) [18]. The occurrence of inactivation varies within the EAG subfamily: while Kv10 (eag) channels show no substantial inactivation, all Kv11 (erg) channels undergo fast C-type inactivation [19], a feature that is otherwise only shared by Kv12.2, but not by Kv12.1 and Kv12.3 elk channels [14]. Inactivating EAG channels pass through the open state during fast recovery from inactivation, which leads to typical biphasic (“hooked”) tail currents when the membrane is repolarized after strong depolarizations [19].

The A371V amino acid substitution in Kv12.2 is located approximately halfway across transmembrane segment S5 (Supplementary Figure S1; [1]), a position not necessarily expected to cause a complete LoF (i.e., diminished surface expression or single-channel conductance). In fact, the previously reported dominant-negative current suppression observed in heteromeric wild-type (WT) + A371V Kv12.2 channels differed for the peak and the sustained current amplitude [1], suggesting that LoF is not only the result of a reduced number of functional channels in the plasma membrane. Therefore, we set out to study the biophysical properties of homomeric and heteromeric Kv12.2 channels containing A371V mutant α-subunits. Our results unveil a mutation-induced drastic enhancement of inactivation as the main mechanism underlying the A371V-mediated Kv12.2 LoF.

## 2. Results

### 2.1. Functional Characterization of A371V Kv12.2 Channels in Xenopus Oocytes

#### 2.1.1. Apparent Complete LoF Caused by the A371V Amino Acid Substitution

The heterologous expression and functional characterization in *Xenopus* oocytes confirmed the previously reported LoF features for A371V Kv12.2 channels. In an external solution with a K^+^ concentration of 5 mM (5 K^+^; see Section 4), resting membrane potential (RMP) measurements yielded values around −40 mV for uninjected oocytes and values close to the K^+^ equilibrium potential (E_K_) of −70 mV for oocytes expressing homomeric WT Kv12.2 channels, indicative of a high expression level of the channels and considerable open probability at negative membrane potentials. Oocytes expressing heteromeric WT + A371V Kv12.2 channels exhibited slightly less negative RMPs. Notably, oocytes expressing only the A371V Kv12.2 mutant exhibited similar RMPs as uninjected oocytes, compatible with a complete LoF (Supplementary Figure S2A).

**Figure 1:**
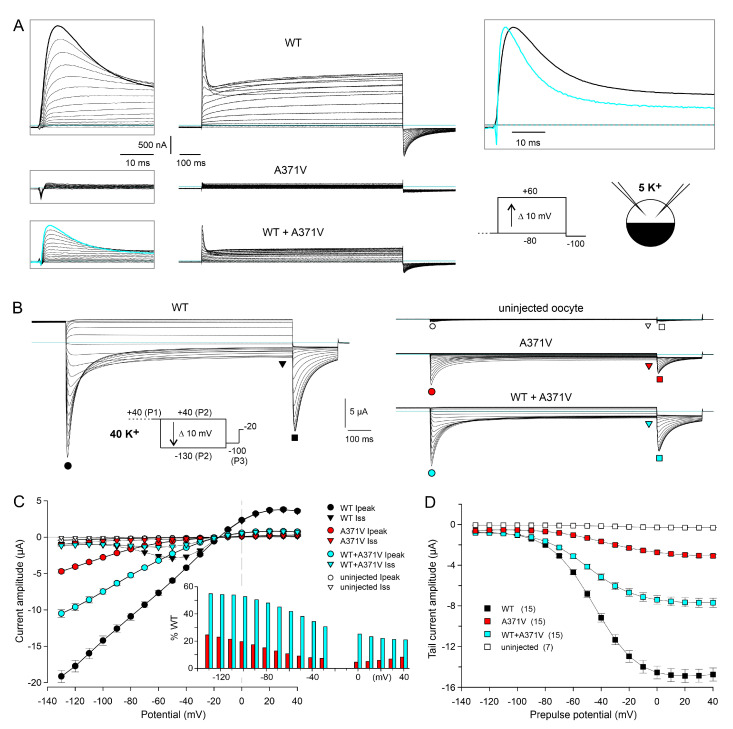
Functional characterization of WT and A371V Kv12.2 channels in *Xenopus* oocytes. (**A**) Two-electrode voltage clamp (TEVC) recordings from *Xenopus* oocytes performed in external solution containing 5 mM K^+^ four days after injection with a total of 5 ng Kv12.2 cRNA (WT only and A371V only), or 2.5 ng of WT and A371V each. Families of current traces were elicited with 1 s depolarizing voltage steps to potentials ranging between −80 and +60 mV to activate the Kv12.2 channels. Boxed left: Currents elicited at the onset of the depolarizing pulses on an expanded timescale to better illustrate the peak currents. Note that, in oocytes expressing the A371V Kv12.2 mutant alone, outward currents and inward tail currents are virtually absent. Boxed upper right: Overlayed current traces elicited with a potential step to +60 mV in oocytes expressing WT (black trace) and WT + A371V Kv12.2 channels (cyan). The current trace recorded from an oocyte co-expressing WT + A371V Kv12.2 was upscaled by a factor of 2.8 to the WT peak amplitude. Horizontal lines indicate zero current. Pulse protocol and recording conditions (TEVC, 5 K^+^) shown in the inset (lower right). (**B**) *Xenopus* oocyte currents recorded in external solution containing 40 mM K^+^ from uninjected control oocytes and from oocytes four days after cRNA injection as in (**A**). Families of current traces were elicited with a triple-pulse protocol. From a depolarized holding potential of −20 mV, a 1 s constant voltage step to +40 mV (P1) was applied to fully activate the channels, followed by variable 1 s test pulses (P2) between +40 and −130 mV, and a final step to −100 mV (P3; see inset). (**C**) Peak and late current amplitudes (means ± SEM; n = 15 different oocytes for each condition; uninjected control oocytes, n = 7) during the variable test pulses. Time point of measurement and meaning of symbols indicated in (**B**). Inset: Mean relative peak current amplitudes of mutant channels relative to WT (%) as a function of the test pulse potential. (**D**) Peak tail current amplitudes (means ± SEM) during the final constant pulse to −100 mV as a function of the preceding variable test pulse potential. Data points of single experiments were fitted with a Boltzmann equation to obtain the parameters of the deactivation curves (see Supplementary Table S1).

Under two-electrode voltage clamp in 5 K^+^, depolarizing pulses from a holding potential of −80 mV to test potentials between −80 and +60 mV elicited typical outward currents in oocytes expressing WT Kv12.2 channels (Figure 1A). With moderate depolarizations, the currents showed slow activation and no macroscopic inactivation, but, with stronger depolarizations (above 0 mV), a transient current component emerged, and the residual current at the end of the test pulse decreased due to the occurrence of voltage-dependent inactivation (Figure 1A, upper panels; Supplementary Figure S2B; [1,20]). Upon hyperpolarization to −100 mV, typical biphasic inward tail currents were recorded, reflecting the passage of channels through the open state during recovery from inactivation before they closed (Figure 1A, upper panels [1,20]). Neither such typical outward currents during depolarizing test pulses, nor such typical inward tail currents at −100 mV were recorded in 5 K^+^ from oocytes expressing only the A371V mutant, also compatible with a complete LoF (Figure 1A, middle panels; Supplementary Figure S2B). However, the co-expression of WT + A371V did result in typical outward and inward currents, and the current amplitudes were disproportionately small (Figure 1A, lower panels; Supplementary Figure S2B,C), indicative of WT + A371V co-assembly with a dominant-negative effect of the mutant, as stated earlier [1]. Moreover, the present data confirm the previously reported different degrees of suppression for the peak and the sustained current component (Supplementary Figure S2C; [1]). Intriguingly, the peak outward currents mediated by heteromeric WT + A371V Kv12.2 channels exhibited accelerated decay kinetics in comparison to homomeric WT Kv12.2 channels (Figure 1A, upper right). Thus, instead of being completely non-functional, A371V mutant α-subunits may induce Kv12.2 channel modulation involving an altered voltage dependence of gating and/or altered kinetics of inactivation.

#### 2.1.2. Elevated External K^+^ Allows the Detection of Tail Currents Mediated by Homomeric A371V Kv12.2 Channels

To investigate whether homomeric A371V Kv12.2 channels are completely non-functional or may exhibit modified gating properties, the concentration of K^+^ in the external solution was raised isotonically to 40 mM (40 K^+^) in order to facilitate channel analysis over a wider voltage range [21]. We assessed the dependence of channel conductance on external K^+^ for Kv12.2 (Supplementary Figure S3), and exemplarily for Kv10.1 and Kv11.1b (Supplementary Figure S4). The resulting data clearly demonstrate nonlinear outward-rectifying instantaneous I–V relationships in asymmetrical K^+^, which correspond well with the predictions of the Goldman–Hodgkin–Katz (GHK) current equation [22] for Kv12.2 and Kv10.1, but an almost linear instantaneous I–V relationship for Kv11.1b (Supplementary Figures S3 and S4; see Section 3).

In addition to elevated external K^+^, we used a triple-pulse protocol (Figure 1B, inset) to test for a functional expression of homomeric A371V Kv12.2 channels. This availability protocol, typically used for Kv11.1 channels to determine the “fully activated” I–V relationship [23], is equally suited for Kv12.2 channels, which share a fast C-type inactivation mechanism with Kv11.1 channels [24]. From a depolarized holding potential of −20 mV, which was close to E_K_ in 40 K^+^, a constant 1 s pulse to +40 mV (P1) served to fully activate and partially inactivate the channels. Then, a variable 1 s test pulse (P2) to potentials ranging from +40 to −130 mV was followed by a final pulse to −100 mV (P3) to elicit tail currents at a fixed potential (Figure 1B). In accordance with a typical feature of the “fully activated” I–V relationship determined with the availability protocol, the peak I–V relationship obtained for WT Kv12.2 channels exhibited a clear inward rectification (Figure 1B,C, black circles). The initial current increase during the variable P2 pulse reflects fast recovery from inactivation, followed by time- and voltage-dependent deactivation at more negative potentials (Figure 1B,C, black triangles). The magnitude of the tail current elicited with the final P3 pulse mirrors the fraction of channels that were still available at the end of the P2 pulse (Figure 1B,C, black squares). The parameters obtained with Boltzmann fits describing the voltage dependence of channel deactivation at the end of the P2 pulses (1 s isochronal deactivation curves) are listed in Supplementary Table S1.

Notably, using the 40 K^+^ external solution combined with the availability protocol allowed for the recording of currents mediated by functionally expressed homomeric A371V Kv12.2 channels (Figure 1B). During test pulses to potentials positive to E_K_ (−20 mV), very small outward currents were recorded, but, during more negative test pulses, transient inward currents became apparent, whose amplitudes clearly exceeded endogenous currents measured in uninjected oocytes from the same donor frog (Figure 1B,C).

#### 2.1.3. Voltage-Dependence of the A371V-Induced LoF Effect

When the fully activated I–V data for homomeric A371V and heteromeric WT + A371V Kv12.2 channels were expressed as the percentage relative to WT, the current-reducing effect of the A371V amino acid substitution was found to be strongly voltage-dependent (Figure 1C, inset). Hyperpolarizing test pulses exerted a facilitating effect on the homomeric A371V currents (4.5% at 0 mV and 23% at −130 mV). The co-expression of WT + A371V resulted in macroscopic currents with amplitudes and characteristics well between those obtained for homomeric WT and homomeric A371V Kv12.2 channels (Figure 1B). Similar to WT, current reversal occurred close to −20 mV, and inward currents were prominent compared to the small sustained outward currents (Figure 1C). In the WT + A371V co-expression experiments, the relative peak current amplitude (in % WT) also tended to increase with more negative test potentials (Figure 1C, inset). However, the percentage was nearly the same at the most negative test potentials (53% at −100 mV and 55% at −130 mV), suggesting that an even stronger hyperpolarization would be unable to yield current levels similar to WT. The inhibiting effect of co-expressed A371V channel subunits increased with less negative potentials and between 0 and +40 mV maximal outward currents amounted to less than 25% of WT (Figure 1C, inset).

The voltage dependence of channel deactivation was assessed by measuring the peak amplitude of the tail currents elicited by the constant P3 pulse to −100 mV of the availability protocol (Figure 1D). Boltzmann fits to tail current amplitudes of the individual experiments yielded moderate but significant differences between the isochronal deactivation of WT and mutant channels. Compared to WT, A371V Kv12.2 channels exhibited a rightward shift in the voltage of half maximal deactivation, and the deactivation curves were less steep for homomeric A371V and heteromeric WT + A371V Kv12.2 channels (Supplementary Table S1).

Functional expression of A371V Kv12.2 channels was demonstrated using oocytes from three different donor frogs, and the consistency of the data confirmed the functional rescue of homomeric A371V and heteromeric WT + A371V Kv12.2 channels by steps to stronger hyperpolarizing potentials starting from an assumed fully activated state (Supplementary Figure S2D). The use of Boltzmann fits to determine the tail current amplitude at −100 mV had the advantage of reducing the impact of unspecific currents and yielded a mean A371V current amplitude of 15 to 20% of the corresponding WT data and a mean relative tail current amplitude of 40 to 50% for heteromeric WT + A371V Kv12.2 channels (Supplementary Figure S2D, left panels). The current-suppressing effect of A371V was significantly stronger at +20 mV (Supplementary Figure S2D, right panels), clearly illustrating the dominant-negative action of the mutant.

Taken together, by adapting the recording conditions, we were able to demonstrate that the *KCNH3* variant A371V does not result in a complete LoF. The magnitude of current suppression by the A371V amino acid substitution in homomeric mutant as well as heteromeric WT + A371V Kv12.2 channels was voltage-dependent, suggesting an effect of the A371V amino acid substitution on the voltage dependence of channel gating.

### 2.2. Functional Characterization of A371V in a Mammalian Expression System

#### 2.2.1. Enhancement in Kv12.2 Channel Expression in CHO Cells by Mild Hypothermia to Analyze the Effect of the A371V Amino Acid Substitution on Channel Availability

For a more detailed analysis of the effect of the *KCNH3* LoF variant A371V on the biophysical properties of the Kv12.2 channel, the *Xenopus* oocyte expression system was of limited usefulness for two major reasons: First, current contamination due to endogenous ion channels often became apparent with the application of strong positive or negative test pulses. These intermingling currents are thought to be mostly Cl^−^ or K^+^ currents [25,26]. Depending on the strain of *Xenopus laevis*, sizable K^+^ currents with inward-rectifying macroscopic features quite similar to Kv12.2 currents can be recorded in external solutions with high K^+^ [27]. Second, *Xenopus* oocytes endogenously express members of the KCNE family of K^+^ channel β-subunits that have been found to interact with heterologously expressed Kv12.2 channels, thereby, reducing their functional expression and shifting their voltage dependence of activation [28].

**Figure 2:**
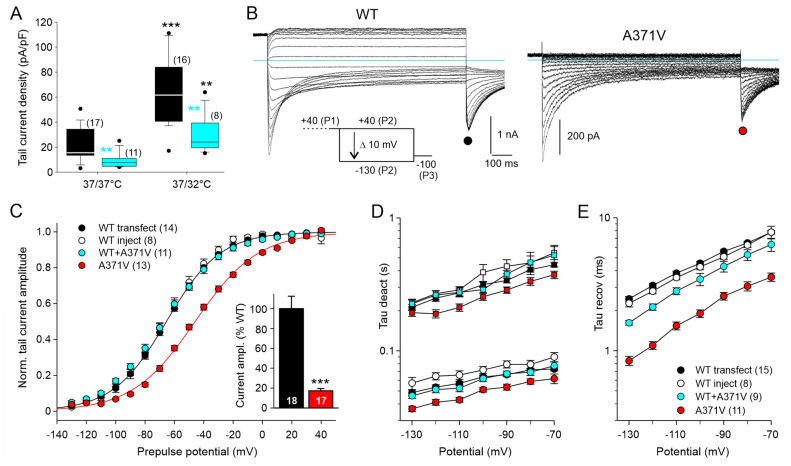
Functional expression of WT and mutant Kv12.2 channels in CHO cells is enhanced by reduced culture temperature enabling biophysical analysis of the LoF mutant. (**A**) Distributions of current densities obtained with two culture conditions (37/37 °C or 37/32 °C) are given as boxplots for WT data (black) and WT + A371V co-expression data (cyan). Electrophysiological experiments were performed 48–60 h after transfecting CHO cells either with WT Kv12.2 channel cDNA alone or with a 1:1 ratio of WT + A371V cDNA, yielding the same total amount of channel cDNA. Part of the glass plates with transfected cells had been transferred to an incubator with a reduced temperature of 32 °C, 20 h after the transfection procedure. Membrane currents were recorded in 40 K^+^ external solution with an availability protocol as shown in (**B**). Tail current amplitudes at −100 mV were obtained from Boltzmann fits to tail current–prepulse voltage relationships. Tail current density was calculated by dividing the current amplitude by the cell capacitance. Number of experiments given in brackets. Black asterisks indicate a significant difference for reduced culture temperature compared to continuous maintenance at 37 °C; cyan asterisks indicate a significant difference between co-expression data and WT data obtained under the same culture condition. ** *p* < 0.01, *** *p* < 0.001; Mann–Whitney U test. (**B**) Membrane currents recorded in CHO cells in 40 K^+^ external solution with the indicated availability protocol three days (37/32/32 °C) after transfection with WT or A371V Kv12.2 cDNA. The holding potential was −30 mV; note the differing current scales. Horizontal lines indicate zero current. (**C**) Normalized tail current amplitudes (means ± SEM) during the final constant pulse to −100 mV are plotted against the preceding variable test pulse potential; prior to averaging, data of individual experiments were normalized using the amplitude of Boltzmann fits to the data points. The black and red lines show Boltzmann fits to the mean normalized data for WT (black) and A371V (red) from transfection experiments. In addition, results from co-expression experiments are given, where single CHO cells were microinjected with WT Kv12.2 cDNA alone as control or with WT + A371V cDNA resulting in a twofold concentration of total channel cDNA. Injected cells were used for recordings two days after injection (32/32 °C). The inset bar plot shows WT and A371V tail current amplitudes (means ± SEM) from the transfection experiments, normalized to the mean WT current amplitude. Number of experiments given in parentheses or at the bottom of the bars. *** *p* < 0.001; unpaired *t*-test. (**D**,**E**) The time course of current traces elicited with test pulses to more negative potentials was fitted with the sum of three exponential functions describing the bi-exponential current decay, yielding a fast (circles) and a slow (squares) time constant of deactivation (**D**), as well as the initial current increase, yielding the time constant of recovery from inactivation (**E**). Color code of symbols and number of experiments given in (**E**) also apply for panel (**D**). Results of statistical analyses of time constants are given in Supplementary Table S1.

In order to circumvent these problems, we switched to CHO cells, which possess almost no voltage-dependent endogenous membrane conductances and are devoid of KCNE β-subunits. Whole-cell patch clamp experiments with WT Kv12.2 confirmed previous studies reporting low heterologous expression levels for this channel in CHO cells [17,20,29]. Even in the 40 K^+^ external solution, specific Kv12.2 currents recorded with the availability protocol two days after cell transfection were usually small, preventing a technically sound comparative analysis of the effect of the A371V LoF variant. We found that the functional expression of WT Kv12.2 channels was significantly enhanced by a reduced temperature during cell culture maintenance of 32 °C for more than one day, as compared to continuous maintenance at 37 °C (Figure 2A). Similar to the results obtained in the *Xenopus* oocyte expression system, parallel experiments with co-transfected A371V resulted in significantly smaller current amplitudes in CHO cells. With both culture conditions, the median current density determined from the tail current amplitudes at −100 mV was about half of the corresponding WT data (Figure 2A), suggesting that the reduced temperature did not relieve the inhibitory effect of A371V co-expression.

We exploited the positive effect of mild hypothermia on functional Kv12.2 channel expression to comparatively analyze homomeric WT and homomeric A371V Kv12.2 channels in CHO cells (Figure 2B). Patch-clamp recordings were performed three days after transfection on cells subjected to the reduced culture temperature one day after transfection. To minimize the cell-to-cell variability in the stoichiometry of heteromeric Kv12.2 channels in co-expression experiments, CHO cells were also microinjected with cDNAs coding for WT and A371V in a fixed 1:1 ratio. The concentration of total channel cDNA was twice the concentration used for the control injection of cells with WT channel cDNA alone to yield more comparable current amplitudes. In this set of experiments, cDNA-injected CHO cells were directly transferred to an incubator with reduced temperature and whole-cell recordings were performed two days after cDNA injection. Two different sets of WT data (transfection and microinjection) served as separate controls for the corresponding parallel experiments on homomeric (transfection) or heteromeric (microinjection) mutant channels. The expression of homomeric WT or A371V Kv12.2 channels in CHO cells yielded distinct current profiles, very similar to the data obtained in *Xenopus* oocytes (Figure 1B and Figure 2B), although half-maximal isochronal deactivation was attained at more negative potentials in CHO cells (Supplementary Table S1). Once more, A371V currents became apparent as small transient inward currents upon stronger hyperpolarizing test pulses, and the P3 tail current amplitude amounted to 17% of the mean WT amplitude (Figure 2C, inset). The isochronal deactivation curve determined for A371V Kv12.2 channels was significantly shifted to more positive potentials by about 20 mV, and A371V Kv12.2 channels exhibited moderately accelerated deactivation kinetics compared to WT channels (Figure 2C,D; Supplementary Table S1). In contrast, heteromeric WT + A371V Kv12.2 channels showed neither a shift in the isochronal deactivation curve nor a significant acceleration of deactivation kinetics (Figure 2C,D). At the onset of the hyperpolarizing pulses, the A371V inward currents steeply increased, indicative of a very fast recovery from inactivation (Figure 2B). Compared to WT, the time constants of recovery from inactivation were significantly smaller by a factor of more than two (Figure 2E; Supplementary Table S1). There was also a tendency towards a faster recovery from inactivation for WT + A371V with significant differences at the most negative test potentials. Most importantly, these experiments in CHO cells confirmed the main findings obtained in the *Xenopus* oocyte expression system, suggesting that the LoF effect of A371V is neither significantly influenced by KCNE β-subunits nor by the temperature during Kv12.2 channel biogenesis.

#### 2.2.2. Moderate Effects of the A371V Amino Acid Substitution on Channel Activation

**Figure 3:**
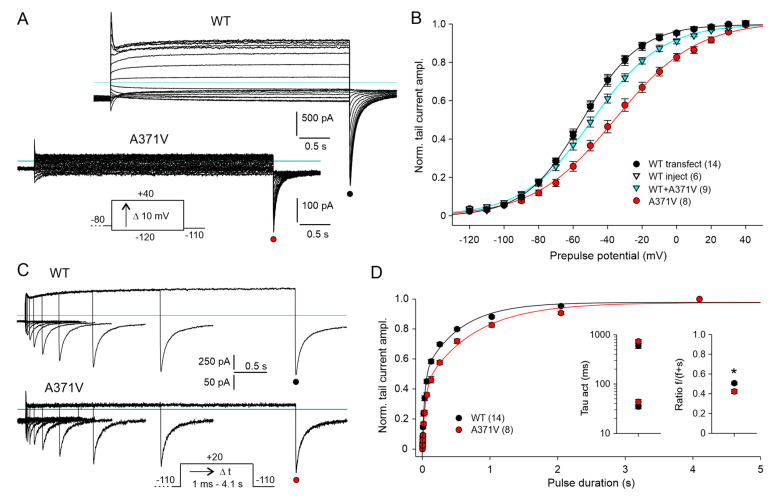
Voltage dependence and time course of WT and mutant Kv12.2 channel activation. (**A**) Membrane currents recorded in CHO cells in 40 K^+^ external solution using an activation protocol three days after transfection with WT or A371V Kv12.2 cDNA. Families of current traces were elicited by hyper- and depolarizing 4 s test pulses to potentials between −120 and +40 mV from a holding potential of −80 mV. The variable test pulses were followed by a final constant potential step to −110 mV to obtain the activation curves from tail current amplitudes. Note the differing current scales. Horizontal lines indicate zero current. (**B**) Normalized tail current amplitudes (means ± SEM) during the final constant pulse to –110 mV are plotted for WT (transfection and injection), A371V (transfection), and co-expression data (injection) against the preceding variable test pulse potential. Prior to averaging, data of individual experiments were normalized using the amplitude of Boltzmann fits to the data points. The lines show Boltzmann fits to the mean normalized data for WT (black), A371V (red), and WT + A371V co-expression (cyan). Number of experiments given in parentheses. (**C**,**D**) The time course of Kv12.2 channel activation was assessed with an envelope-of-tails protocol. (**C**) Overlays of WT and A371V current traces recorded with a triple-pulse protocol consisting of a constant hyperpolarizing pulse to −110 mV, a test pulse to +20 mV of increasing duration, and a final pulse to −110 mV to elicit the tail currents. (**D**) Normalized tail current amplitudes (means ± SEM) plotted against the duration of the depolarizing pulse to +20 mV. The lines show double exponential fits to the data. Means ± SEM for the two time constants and for the relative amplitude of the fast activating current component, respectively, are given in the left and right inset; * *p* < 0.05. Mean values and results of statistical data analyses are given in Supplementary Table S1.

Next, we studied the effect of the A371V amino acid substitution on Kv12.2 channel activation. The voltage dependence of channel activation was determined from tail currents elicited by a hyperpolarization to −110 mV following 4 s test pulses to potentials between −120 and +40 mV (Figure 3A, inset). WT channel activation occurred over a broad voltage range with prominent activation already at negative potentials (Figure 3A,B).

Similar to the voltage dependence of deactivation, half-maximal activation of A371V Kv12.2 channels was shifted by about 15 mV to more positive potentials, and the steepness of the activation curve was decreased (Figure 3B; Supplementary Table S1). The co-expression of WT + A371V yielded an activation curve between those of the corresponding homomeric channels. There was no significant change in the voltage for half-maximal activation, but the activation curve was flattened, pointing to a lower voltage sensitivity (Figure 3B; Supplementary Table S1).

The effect of the A371V amino acid substitution on the kinetics of Kv12.2 channel activation was tested at +20 mV with an envelope-of-tails protocol (Figure 3C). The increase in tail current amplitude as a result of prolonged test pulses had to be fitted with the sum of two exponential functions, yielding a fast and a slow time constant of activation for both WT and mutant (Figure 3D). Time constants determined for A371V Kv12.2 channels were not significantly larger, but the ratio of the fast-activating current component was slightly smaller for A371V and WT + A371V compared to the corresponding WT data (Figure 3D; Supplementary Table S1). In summary, the A371V amino acid substitution exerted moderate effects on the voltage dependences and kinetics of activation and deactivation of the homomeric A371V Kv12.2 channels. These effects were mitigated or even absent in the heteromeric WT + A371V Kv12.2 channels.

#### 2.2.3. The A371V Amino Acid Substitution Drastically Enhances Channel Inactivation

**Figure 4:**
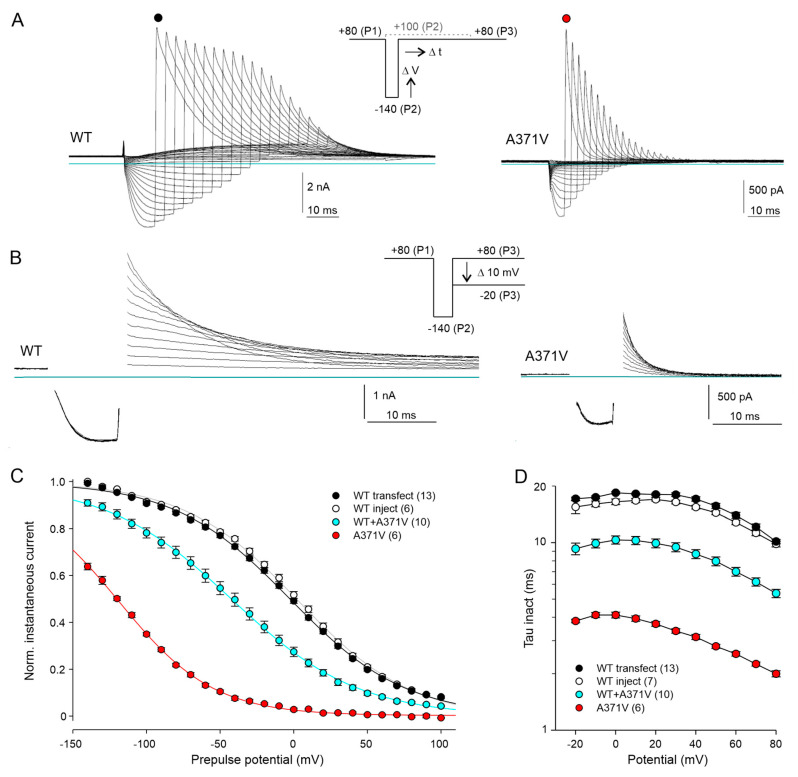
The A371V amino acid substitution drastically affects voltage dependence and time course of Kv12.2 channel inactivation. (**A**) Overlays of WT and A371V Kv12.2 current traces recorded in CHO cells in 40 K^+^ external solution with the indicated triple-pulse protocol. From a holding potential of −30 mV, a first pulse to +80 mV (P1) to activate and inactivate the channels was followed by a step to potentials between −140 and +100 mV of variable duration (P2) to induce recovery from inactivation with minimal deactivation. A final pulse to +80 mV (P3) reinitiated inactivation. P2 duration steadily increased with more positive potentials and differed for WT and mutant Kv12.2 channels to account for significant differences in the rate of recovery from inactivation (see Figure 2E). (**B**) Families of WT and A371V Kv12.2 current traces recorded with a slightly modified triple-pulse protocol. From a holding potential of −30 mV, a first pulse to +80 mV (P1) was followed by a constant step to −140 mV (P2) to induce recovery from inactivation in most channels. The variable P3 pulse to potentials between +80 and −20 mV served to assess the inactivation rate. Once more, P2 duration differed for WT and mutant Kv12.2 channels. Capacitive transients are blanked out for clarity. Horizontal lines indicate zero current. (**C**) Normalized P3 peak currents (means ± SEM) from experiments as shown in (**A**) as a function of the P2 potential. Current amplitudes were measured directly after the capacitive current transient and normalized prior to averaging using Boltzmann fits to the individual data. Plotted lines show Boltzmann fits to mean values and represent inactivation curves. (**D**) Time constants of inactivation (means ± SEM) determined from experiments as shown in (**B**) by fitting a single exponential function to the P3 current decay after the capacitive current component. Data points connected by straight lines; number of experiments given in parentheses. Results of statistical data analyses are given in Supplementary Table S1.

Thus far, no specific A371V outward currents were recorded with the applied protocols, suggesting almost complete inactivation of the mutant Kv12.2 channels at depolarized potentials. To assess the voltage dependence of Kv12.2 channel inactivation, we used a triple-pulse protocol (Figure 4A, inset), starting with a P1 pulse to +80 mV to fully activate and inactivate the channels. Variable P2 pulses to potentials between −140 and +100 mV served to induce voltage-dependent recovery from inactivation, and a constant P3 pulse back to +80 mV caused re-inactivation of open channels. The relative amplitude of the transient P3 currents mirror the fraction of channels, which have opened during the P2 pulse and have not deactivated yet. To account for the strong voltage dependence of the kinetics of recovery from inactivation (see Figure 2E), the duration of the P2 pulse was steadily increased with more positive pulse potentials. The duration of the most negative pulses was adapted to minimize deactivation and thus maximize the number of open channels. For all potentials, the P2 pulses used in the A371V experiments were shorter than for those WT experiments due to the much faster recovery from inactivation of the mutant channels (see Figure 2E).

With this protocol, exponentially decaying WT and A371V outward currents became visible at the start of the P3 pulse (Figure 4A). The amplitude of the A371V current transient was maximal after the most negative P2 pulse (−140 mV) and decreased steadily with less negative P2 pulses (Figure 4A, right panel). Inactivation kinetics were determined with a slightly different triple-pulse protocol, in which a short constant P2 pulse to −140 mV to re-open inactivated channels was followed by variable P3 pulses from +80 to −20 mV (Figure 4B). The strong impact of the A371V amino acid substitution on voltage- and time-dependent Kv12.2 channel inactivation is illustrated in Figure 4C,D. Half-maximal steady-state inactivation occurred at around 0 mV in WT Kv12.2 channels (Figure 4C) and inactivation had a low voltage sensitivity, indicated by the shallow inactivation curve with a slope factor *k* of almost 40 mV (Figure 4C; Supplementary Table S1).

Notably, in homomeric A371V channels, the voltage dependence of inactivation was shifted by 120 mV to more negative potentials (Figure 4C) and inactivation kinetics were accelerated by a factor of about five (Figure 4D; Supplementary Table S1). With co-expressed WT + A371V channel subunits, channel inactivation was still strongly enhanced with a shift in the steady-state inactivation curve by about 40 mV to more negative potentials (Figure 4C) and significantly faster inactivation kinetics compared to homomeric WT channels (Figure 4D; Supplementary Table S1).

**Figure A1:**
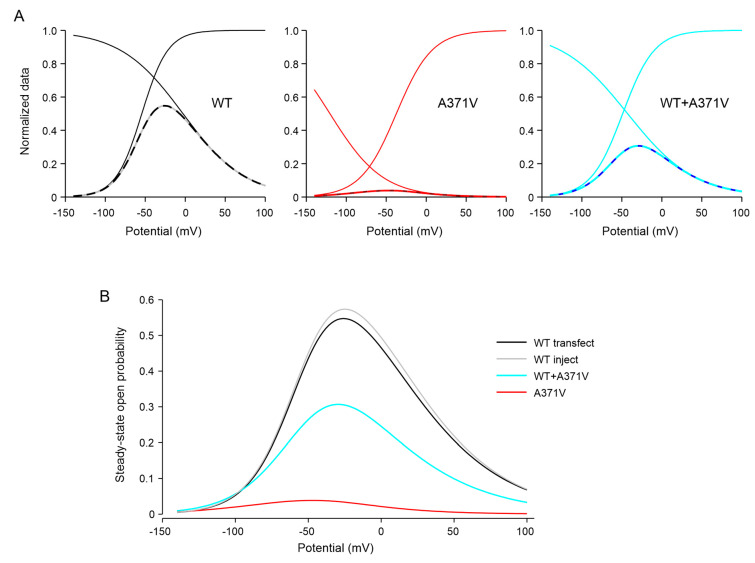
Window curves of functionally expressed homomeric WT, homomeric A371V, and heteromeric WT + A371V Kv12.2 channels. (**A**) Steady-state channel open probability curves (broken lines) were obtained by multiplying the respective activation and inactivation curves defined by the means of the Boltzmann parameter *V*_1/2_ and *k* determined in CHO cells in external solution containing 40 mM K^+^ (see Supplementary Table S1). (**B**) Overlay of window curves. WT Kv12.2 channels exhibit considerable steady-state open probability in the whole range of physiological membrane potentials with a maximal value of 0.56 at −25 mV. Co-expression of A371V yielded a maximum of 0.3 at −30 mV and homomeric A371V channels exhibit only marginal open probability with a maximum of 0.038 at −48 mV.

Taken together, we show that, in addition to a rather moderate effect on channel activation, by far the most prominent effect of the A371V amino acid substitution on the biophysical properties of homomeric as well as heteromeric mutant Kv12.2 channels is the strongly enhanced inactivation. Combining the effects on the voltage dependences of activation and inactivation by multiplying the data underlying the respective activation (see Figure 3B) and inactivation curves (see Figure 4C) of the different Kv12.2 channels (homomeric WT, homomeric A371V, and heteromeric WT + A371V) yielded steady-state open probability window curves (Appendix A, Figure A1), impressively illustrating the cause of the apparent complete LoF of A371V channels (see Section 3).

## 3. Discussion

In the present study, we confirm the previously reported apparent complete LoF for homomeric A371V mutant Kv12.2 channels [1]. In addition to the heterologous expression of channels in *Xenopus* oocytes, we have utilized CHO cells combined with transient cDNA transfection and microinjection and with variable cell maintenance temperatures. By adapting the recording conditions, especially by elevating the external K^+^ concentration (40 mM), we detected specific homomeric A371V Kv12.2 channel-mediated currents in *Xenopus* oocytes as well as in CHO cells. Further experiments in both expression systems, addressing the external K^+^ dependence of Kv12.2 channels, suggested that two effects of increasing external K^+^ enabled the functional characterization of homomeric A371V Kv12.2 channels: the strong increase in K^+^ conductance at negative potentials (Supplementary Figure S3) and a marked slowing of inactivation kinetics (Supplementary Figure S5). These external K^+^ effects on the biophysical properties of Kv12.2 channels will be addressed in more detail below.

**Heterologous expression of WT and A371V mutant Kv12.2 channels**

Our analyses of Kv12.2 channel-mediated current recordings in elevated external K^+^ yielded very similar effects of the A371V mutant in *Xenopus* oocytes and CHO cells. This shows that K^+^ channel β-subunits of the KCNE family, which are endogenously expressed in *Xenopus* oocytes [28] but not in CHO cells, do not significantly alter the effect of the A371V amino acid substitution. Nevertheless, the association of KCNE subunits might have caused the observed difference in the voltage dependence of Kv12.2 channel deactivation, determined in *Xenopus* oocytes and CHO cells (Supplementary Table S1). In fact, endogenous *Xenopus* and heterologous mouse KCNE1 and KCNE3 β-subunits have been shown previously to interact with melk2 (mouse Kv12.2) to reduce the functional channel expression and shift the voltage dependence of activation to more depolarized potentials [28]. However, these expression-system-related differences are negligible, given the prominent effects of the A371V variant to be discussed in the context of gating modulation.

**Impact of the A371V amino acid substitution on Kv12.2 channel gating properties**

The A371V amino acid substitution induced moderate but significant effects on channel activation and deactivation. The steady-state voltage dependence was shifted to more positive potentials and the voltage sensitivity was reduced. Moreover, channel deactivation was accelerated. Analysis of channel activation kinetics yielded a fast and a slowly activating current component. The slow time constant might be related to the phenomenon of the voltage-dependent potentiation or “mode shift” described for human Kv12.1 [30] and the zebrafish Kv12 channel [31], similar to Kv11.1 [32]. We found that not only homomeric A371V, but also heteromeric WT + A371V Kv12.2 channels exhibited a reduced fraction of the fast-activating current component. This effect probably contributed to the significant reduction in the depolarization-induced initial peak current amplitude determined with physiological external K^+^ for heteromeric WT + A371V Kv12.2 channels.

Although the effects on activation and deactivation are likely to contribute to current suppression, the effect of the A371V amino acid substitution on Kv12.2 channel inactivation was much more pronounced: the voltage dependence of steady-state inactivation was shifted by about 120 mV to more negative potentials, resulting in an almost complete suppression of A371V currents in the physiological range of membrane potentials. In addition, transitions between the open and the inactivated state were strongly accelerated. Of note, the properties of heteromeric WT + A371V Kv12.2 channels were well in between those of the respective homomeric channels: in the co-expression experiments, the steady-state inactivation curve remained shifted by about 40 mV to more negative potentials and the time constants of inactivation were nearly half of the WT values. Considerably accelerated inactivation kinetics result in a marked suppression of outward current by severely limiting the amount of time channels stay open upon depolarization, as shown for the Kv11.1 (HERG) channel [33]. Interestingly, heteromeric WT + A561V HERG channels are also reported to exhibit enhanced inactivation with a leftward shift in the inactivation curve by about 40 mV [11].

Using the normalized steady-state activation and inactivation curves, steady-state channel open probabilities were calculated as a function of the membrane potential. While substantial steady-state activity of WT Kv12.2 channels occurred in the whole range of physiological membrane potentials, A371V Kv12.2 channels exhibited only marginal steady-state open probability (Appendix A, Figure A1). Our WT data have confirmed that human Kv12 channels tend to activate at more negative values compared to their rat or mouse Kv12 counterparts [1,20,21,24,34], resulting in an extremely broad open probability window with a remarkable maximum above 0.5 for human Kv12.2 channels (Appendix A, Figure A1), making these channels ideal candidates for the suppression of neuronal activity at subthreshold potentials in the human brain.

**Possible effects of the A371V amino acid substitution on Kv12.2 trafficking**

Reduced current amplitudes and, thus, LoF of a voltage-gated ion channel may result from additive effects due to gating modulation and impaired trafficking. Our present co-expression data suggest such an additive effect, because outward current amplitudes were more strongly suppressed than channel open probability: sustained WT + A371V outward current amplitudes at +20 mV amounted to about 25% (see Supplementary Figure S2C,D), whereas steady-state open probability was about 50% of homomeric WT channels in this voltage range (Appendix A, Figure A1). In addition, even strong hyperpolarizations failed to completely rescue the tail current amplitudes of fully activated WT + A371V channels (Figure 1C, inset). Thus far, the low expression level of Kv12.2 channels in CHO cells (this study and [17,20,29]) impeded comparative studies on WT and mutant Kv12.2 channel trafficking. Insufficient glycosylation of extracellular N-glycosylation sites located in the long S5/P linker domain (see Supplementary Figure S1) is suggested to underlie the low functional Kv12.2 channel expression in mammalian heterologous expression systems [17].

Remarkably, the analogous LQT2 S5 variant A561V in *KCNH2* has also been found to result in complete loss of HERG channel function [12]. A trafficking defect, that was shown to be neither rescued by channel blocking agents nor by hypothermia during biogenesis, could explain the LoF of A561V in HERG [35]. Nevertheless, a partial rescue of the trafficking defect by low temperature was described for heteromeric WT + A561V HERG channels [36]. Here, we strongly enhanced the functional expression of Kv12.2 channels in CHO cells by mild hypothermia; however, there was no indication of a stronger increase in current density for heteromeric WT + A371V compared to homomeric WT Kv12.2 channels. In the context of large-scale gene expression, it is well established that CHO cells respond to mild hypothermia with an increase in protein production after transient transfection [37]. The low heterologous expression level of Kv12.2 channels has led to Kv12.2 being regarded as a “silent” channel [29]. This might also be one of the reasons for the paucity of electrophysiological studies on this channel. The strongly increased Kv12.2 channel expression in CHO cells by mild hypothermia can facilitate future studies on Kv12.2.

**The K^+^ dependence of Kv12.2-mediated conductance**

The external K^+^ concentration plays a crucial role in determining the electrical activity of excitable cells, and increases in external K^+^ are clearly associated with epileptiform activity [38]. Since most of our present electrophysiological experiments were performed with elevated external K^+^, we also investigated the K^+^ dependence of Kv12.2-mediated channel conductance. Using availability protocols, we were able to record specific A371V currents in high external K^+^ as transient inward currents (Figure 1B and Figure 2B). Current amplitudes increased with more negative potentials, which is partially explained by the increase in driving force. The modified Ohm’s law is often used to define the factor of the driving force as “E-E_K_” to calculate channel current directly from K^+^ channel open probability or vice versa. This simple procedure assumes a voltage-independent channel conductance (i.e., no GHK rectification [22,39]). Indeed, this feature has been shown for all Kv11 subfamily members with the demonstration of linear instantaneous I–V relationships despite asymmetrical intra- and extracellular K^+^ concentrations [33,40,41,42]. Here, we found a clear difference in the properties of Kv12.2 compared to Kv11 channels, as Kv12.2 channels almost perfectly obey the predictions of the GHK current equation (Supplementary Figure S3). The GHK current equation implies that ions can flow more easily from a compartment with a high concentration to a compartment with a low concentration than in the opposite direction, resulting in a decrease in single-channel conductance at negative potentials in physiological K^+^ gradients [22]. We describe the GHK behavior of Kv12.2 channels in *Xenopus* oocytes (Supplementary Figure S3A,B) as well as in CHO cells (Supplementary Figure S3C) and confirm the contrasting almost voltage-independent conductance of Kv11.1 channels using the Kv11.1b isoform expressed in CHO cells (Supplementary Figure S4B,C).

The GHK equation has previously been used to calculate voltage-dependent Kv10.1 channel activation from classical I–V data [39,43,44,45]. Our present comparative data directly demonstrate nonlinear outward-rectifying instantaneous I–V relationships in asymmetrical K^+^ for Kv10.1 channels (Supplementary Figure S4A,C). As Kv10.1 channels normally deactivate very rapidly in the negative potential range, two mutant Kv10.1 channels were utilized in these experiments. These mutants had previously been shown to activate and deactivate at considerably more negative potentials and to exhibit strongly decelerated deactivation kinetics [44], enabling a more accurate determination of instantaneous current amplitudes (Supplementary Figure S4A).

It has previously been shown that elevating the external K^+^ concentration results in a strong increase in Kv12.2 channel conductance in the negative voltage range [21], and it was supposed that Kv12.2 channel conductance linearly increases with external K^+^. Our data confirm this behavior at negative potentials, but they show that the K^+^-dependent conductance increase cannot be extrapolated to positive potentials due to the GHK behavior of Kv12.2 channels. Nevertheless, our paired experiments in oocytes indicated an additional voltage-independent increase in Kv12.2 channel conductance of about 30% in 40 mM K^+^ compared to 5 mM external K^+^, resulting in increased instantaneous outward current amplitudes at strong positive potentials (Supplementary Figure S3A,B).

In HERG and the other members of the Kv11 subfamily, channel conductance is roughly proportional to the square root of the external K^+^ concentration independent of the actual membrane potential [42,46,47]. Thus, compared to Kv12.2, Kv11 channels exhibit a lower sensitivity to external K^+^ at negative potentials, but a higher sensitivity to changes in external K^+^ at depolarized potentials. When measuring Kv12.2 currents in low external K^+^, the reduced Kv12.2 single-channel conductance at negative potentials can easily lead to an underestimation of the number of open Kv12.2 channels in the negative voltage range. However, one may speculate that a fraction of Kv12.2 channels, which are open at the RMP of a neuron, can efficiently dampen cellular excitability, since depolarizing stimuli are counteracted by instantaneous increases in channel conductance. On the other hand, increases in external K^+^ may strongly enhance the resting Kv12.2 conductance, clamping the resting potential close to the K^+^ equilibrium potential.

**Fast C-type inactivation and structural impact of the A371V amino acid substitution in Kv12.2**

Kv12.2 channels are thought to undergo C-type inactivation analogous to HERG channels. Experimental evidence of Kv12.2 C-type inactivation is based on the slowing of inactivation kinetics by external quaternary ammonium ions, as well as on mutational analysis, demonstrating that analogous single amino acid substitutions in Kv12.2 and HERG similarly interfere with channel inactivation [24]. Despite these similarities, half-maximal steady-state inactivation of Kv12.2 channels occurs at around 0 mV (present data and [21,24]), which is considerably more positive compared to HERG channels where half-maximal inactivation occurs around −70 mV [40,48,49]. This large difference has been attributed to positively charged amino acid residues in the turret helix of Kv12.2 (Supplementary Figure S1A; [49]).

An inherent voltage dependence of inactivation, which is independent of voltage-dependent channel activation, is regarded to be a special feature of HERG C-type inactivation [33,50]. Notably, a similar inherent voltage dependence of inactivation in Kv12.2 channels is supported by the effects of the A371V amino acid substitution, which drastically affects the voltage dependence of inactivation with comparably moderate effects on the voltage-dependence of activation. In addition, we show that the time course of WT Kv12.2 channel inactivation is significantly slowed by an increase in external K^+^ (Supplementary Figure S5), which is another typical feature of C-type inactivation in Kv11 channels [42,50]. In fact, our data strongly suggest that this slowing of inactivation in elevated external K^+^ enabled the visualization and analysis of homomeric mutant A371V Kv12.2 channel inactivation.

Although homomeric A561V HERG channels do not mediate recognizable currents [11,12], suppositions regarding the mechanism of enhanced inactivation in A371V Kv12.2 channels may be derived from a structural analogy to the HERG channel (see Supplementary Figures S1A and S6) and information from the functional effects of natural or specifically designed HERG point mutations which affect channel inactivation [51]. Fast voltage-dependent inactivation of HERG channels is suggested to involve a complex interplay between parts of the voltage sensor (S4 and S1) and helical structures of the outer pore region (S5, P, T; Supplementary Figure S1), from where conformational changes are transferred to the selectivity filter [51,52]. We have explored the structural impact of the A371V amino acid exchange with Kv12.2 homology modeling based on the structural coordinates of the HERG channel (Supplementary Figure S6; [53]). This modeling approach suggests that the A371V amino acid substitution occurs at a site close to the pore helix of the same Kv12.2 α-subunit. The closest distance (3.6 Å) is suggested for an opposing Leu460 located in the pore helix (Supplementary Figure S6A). Replacement of Ala371 by the larger valine is predicted to result in a moderate alteration of that distance (3.7 Å) combined with a displacement of the side chain of Leu460. Distance alterations were also predicted between Val371 and the side chains of other relevant amino acids in the pore helix, including Leu456, Ala459, and Leu463 (Supplementary Figure S6B). These alterations may potentially influence the gating of mutant Kv12.2 channels. In HERG channels, this region of tight interaction between S5 and the pore helix is suggested to involve the small side chains of Ala561 and Ala565 in S5, which slot between side chains of the pore helix [52]. This region of the pore helix is not completely conserved among EAG channel subtypes, and the Kv12.2 pore helix amino acids L456, A459, L460, and L463 correspond to HERG L615, T618, F619, and L622 (Supplementary Figure S1A). All of these amino acids were implicated in HERG channel inactivation gating [54]. T618A HERG channels exhibit strongly accelerated inactivation, whereas the F619L exchange exerted no remarkable effect on HERG inactivation gating, which is in sharp contrast to several other amino acid exchanges at this position [54].

Intriguingly, the aromatic amino acid F619 (Supplementary Figure S1A) has been identified as part of the binding site for special small-molecule HERG activators, which impair channel inactivation [55,56]. In recent years, huge efforts have been made to develop such small-molecule “HERG activators” to treat cardiac LQT syndromes by mechanism-based pharmacotherapy [57]. In a similar fashion, the treatment of epilepsy is currently undergoing a paradigm shift from symptom-only anti-seizure medications to a mechanism-based treatment of specific epilepsy syndromes [58].

The substance ICA-105574 is known to increase HERG outward currents mainly by shifting the voltage dependence of channel inactivation to more positive potentials [59]. Interestingly, ICA-105574 has also been found to bind to and inhibit Kv10.1 channels (which lack C-type inactivation), despite several differences in the amino acids constituting the putative drug binding pocket in HERG [43,60]. We have now investigated whether ICA-105574 would be able to inhibit C-type inactivation in Kv12.2 channels using experimental conditions where HERG outward currents were clearly increased (Supplementary Figure S7B,D), but we found that Kv12.2 remained virtually unaffected by ICA-105574 (Supplementary Figure S7A,C).

Our present study reveals the demand for a pharmacological substance that specifically affects Kv12.2 channel inactivation and could counteract the drastically enhanced inactivation present in mutant A371V channels. In addition, more information on the role of Kv12.2 channels in neuronal activity and the impact of LoF mutations in the maturing and the adult human brain will be needed to estimate the capability of such a mechanism-based treatment in order to ameliorate neurological and psychiatric phenotypes associated with LoF variants like the one studied herein [1].

## 4. Materials and Methods

**Heterologous channel expression**

Kv12.2 channels were expressed in *Xenopus laevis* oocytes and Chinese hamster ovary (CHO) cells. Oocytes were collected from female frogs under tricaine anesthesia. All experimental procedures were in accordance with the national guidelines for the care and use of research animals and were approved by the local authorities. The follicular tissue was removed by collagenase treatment. Before and after injection of cRNA, oocytes were kept at 16 °C in medium containing 75 mM NaCl, 5 mM sodium pyruvate, 2 mM KCl, 2 mM CaCl_2_, 1 mM MgCl_2_, and 5 mM HEPES; pH adjusted to 7.5 with NaOH; medium supplemented with 50 mg/L gentamicin. *Xenopus laevis* oocytes were injected with cRNA encoding either human Kv12.2 wild-type (WT; 5 ng/50 nL) channel or the A371V mutant (5 ng/50 nL) [1] using a Nanoliter 2000 microinjector (World Precision Instruments, Berlin, Germany). For co-expression of WT and mutant, the 50 nL injection volume contained 2.5 ng of each cRNA. Oocytes were used for experiments 3–5 days after injection.

CHO cells were transfected with WT or mutant cDNA encoding human Kv12.2 (see Supplementary Methods in [1]) and with cDNA encoding EGFP-N1 (Clontech, Mountain View, CA, USA), using Lipofectamine 2000 (Invitrogen, Waltham, MA, USA). The final concentration of channel cDNA cloned into the pcDNA3.1 vector was mostly 1.5 µg/mL. Only for WT and A371V co-expression studies by cell transfection, 0.5 µg/mL of each plasmid was used with parallel WT transfection experiments (1.0 µg/mL). For co-expression experiments of WT and A371V with a fixed intracellular cDNA ratio, CHO cells were microinjected with a FemtoJet 4i (Eppendorf, Hamburg, Germany) with equal amounts of both channel-coding cDNA plasmids (25 ng/µL each), together with EGFP-N1 as described previously [44,61]. Control experiments were performed with WT channel cDNA (25 ng/µL) microinjected in parallel.

If not stated otherwise, transfected CHO cells were subjected to mild hypothermia (32 °C) the following day and electrophysiological experiments were performed 3 days after cell transfection (37/32/32 °C). Microinjected CHO cells were directly subjected to the reduced culture temperature and recorded two days after microinjection (32/32 °C).

**Electrophysiology**

*Xenopus* oocyte currents were recorded under two-electrode voltage clamp using a Turbo TEC-10CX amplifier (npi electronic GmbH, Tamm, Germany) and PATCHMASTER acquisition software (version 2x73.5; HEKA Elektronik, Reutlingen, Germany). Oocytes were bathed in a 5 mM K^+^ solution (5 K^+^) containing 91 mM NaCl, 5 mM KCl, 1 mM CaCl_2_, 1 mM MgCl_2_, 5 mM HEPES, or in a 40 mM K^+^ solution (40 K^+^) containing 56 mM NaCl, 40 mM KCl, 1 mM CaCl_2_, 1 mM MgCl_2_, 5 mM HEPES; pH adjusted to 7.4 with NaOH. Pipettes were filled with a 3 M KCl solution. Experiments were performed at room temperature.

Patch clamp experiments were performed on individual CHO cells in the conventional whole-cell configuration. The external Ringer solution (5 mM K^+^) contained 140 mM NaCl, 5 mM KCl, 0.8 mM MgCl_2_, 1 mM CaCl_2_, 5 mM glucose, 10 mM HEPES, and pH adjusted to 7.4 with NaOH. The standard high K^+^ (40 mM) external solution contained 105 mM NaCl, 40 mM KCl, 0.8 mM MgCl_2_, 1 mM CaCl_2_, 5 mM glucose, and 10 mM HEPES; pH adjusted to 7.4 with NaOH. The pipette solution contained 125 mM potassium aspartate, 16 mM KCl, 2 mM MgCl_2_, 2 mM CaCl_2_, 10 mM HEPES, 5 mM EGTA, 5 mM K_2_-ATP, and pH adjusted to 7.2 with KOH. Patch pipettes had resistances of 3.0 to 4.5 MΩ when filled with intracellular solution. Data were online-corrected for a liquid junction potential of about −13 mV for an aspartate-based intracellular solution; no leak subtraction was performed. Series resistance compensation was as high as possible (60 to 90%). Signals were compensated for both fast and slow capacity transients, low-pass filtered at 10 and 3 kHz, and digitized at sample intervals of 50 µs (inactivation protocols) to 1 ms (activation protocols). An EPC-9 patch clamp amplifier was used in combination with the PATCHMASTER stimulation and data acquisition software (version 2x90.1; HEKA Elektronik, Reutlingen, Germany). Pulse protocols are depicted in the figures. The experiments were performed at room temperature.

**Data analysis and statistics**

Data processing was performed with FITMASTER (version 2x73.2; HEKA Elektronik, Reutlingen, Germany), Excel (Microsoft Corp., Redmond, WA, USA), and SigmaPlot 11.0 and 13.0 (Systat Software, Düsseldorf, Germany). Kv12.2 tail current-voltage (G/V) relationships were analyzed with a first-order Boltzmann function of the form:

$$\frac{G}{G_{max}}=\frac{a}{\left(1+\exp(-\frac{\left(V-V_{\frac{1}{2}}\right)}{k})\right)}+b$$

with *V*_1/2_ as the potential of half-maximal conductance and *k* as the slope factor for the specific Kv12.2 current component with amplitude *a*, and *b* as the amplitude of the residual unspecific current component.

Most experimental data are given as means ± SEM, with *n* representing the number of experiments from different cells. For multiple comparisons of data obtained in *Xenopus* oocytes, one-way ANOVA with post hoc Bonferroni’s test (all pairwise multiple-comparison procedure; Supplementary Figure S2) or post hoc Dunnett’s test (comparison to a single control group; Supplementary Table S1) was used to test for significance. Most data obtained in CHO cells were analyzed by Student’s two-tailed unpaired *t*-test. Current density values showed high variability and some of the data groups failed to pass the Shapiro–Wilk normality test. These data are presented as box plot (Figure 2A; Cleveland method; boxes indicate the 25 to 75% range, and whiskers and points indicate the 10/90 percentiles and the 5/95 percentiles, respectively; the median is shown as line). Statistical analysis of current density was performed with the Mann–Whitney U test. In all statistical analyses, significance level was set to 0.05, and statistical significance is indicated by * *p* < 0.05, ** *p* < 0.01, and *** *p* < 0.001. Statistical testing was performed with SigmaPlot 11.0 and SigmaPlot 13.0.

## 5. Conclusions

We have characterized Kv12.2 channels and the effects of the recently identified *KCNH3* variant c.1112C>T; p.(Ala371Val), which is associated with seizures and additional severe neurophysiological symptoms. We confirm LoF of A371V channels and the dominant-negative action of A371V upon co-expression with WT, and demonstrate that an apparent complete LoF results from a dramatic enhancement in channel inactivation.

Our co-expression experiments clearly show that the decreased Kv12.2 K^+^ conductance is not merely due to fewer channels in the plasma membrane (as in heterozygous *Kcnh3* knockout mice), although our data suggest that this parameter contributed to the observed reduction in current amplitude.

The extremely broad open probability window of human Kv12.2 channels combined with their reported wide and significant expression in the brain suggests Kv12.2 as an important subthreshold channel controlling neuronal activity. In contrast to classical inward rectifier channels (or Kv11 channels), the GHK-rectification property limits Kv12.2 conductance at the RMP in low external K^+^, possibly avoiding excessive K^+^ loss or excessive inhibition of excitability.

It may be speculated that the heterozygous A371V variant in *KCNH3* may result in a depolarization of the RMP, a lowered threshold for action potential generation, and either induction or inhibition of neuronal high-frequency firing due to strongly enhanced and accelerated Kv12.2 channel inactivation. Increased excitability is a key feature in the pathophysiology of epilepsy, and altered neuronal activity in different regions of the brain may result in diverse neurophysiological symptoms. The elucidation of the effects of a gene variant on the biophysical properties of the encoded ion channel is a prerequisite for the future putative development of mechanism-based treatments of channelopathies.

## Data Availability

All study data are included in the article and the Supplementary Materials.

## Supplementary Materials

The following supporting information can be downloaded at: https://www.mdpi.com/article/10.3390/ijms26104631/s1, Supplementary Figures S1–S7 and Supplementary Table S1.

## Abbreviations

The following abbreviations are used in this manuscript:

WT	wild-type
E_K_	K^+^ equilibrium potential
LoF	loss of function
GHK	Goldman–Hodgkin–Katz
RMP	resting membrane potential
CHO	Chinese hamster ovary
