# Supplementary material for: KCNH3 Loss-of-Function Variant Associated with Epilepsy and Neurodevelopmental Delay Enhances Kv12.2 Channel Inactivation
Source: Int J Mol Sci. 2025 May 13;26(10):4631. doi: 10.3390/ijms26104631 (PMC12111102; doi:10.3390/ijms26104631)
Supplement: Supplementary file 1 [file ijms-26-04631-s001.zip › ijms-3609363-supplementary.pdf]

Supplementary Materials for

KCNH3 loss-of-function variant associated with epilepsy and neurodevelopmental delay enhances Kv12.2 channel inactivation

Christiane K. Bauer[1*], Arne Bilet[1], Frederike L. Harms[2] and Robert Bähring[1*]

[1] Institute of Cellular and Integrative Physiology, University Medical Center Hamburg-Eppendorf, Hamburg, 20246, Germany
[2] Institute of Human Genetics, University Medical Center Hamburg-Eppendorf, Hamburg, 20246, Germany
* Correspondence: c.bauer@uke.de, r.baehring@uke.de

This file includes Supplementary Figures S1-S7 and

Supplementary Table S1

A

```
                                            S5                               T
Kv10.1  KCNH1  eag1  DHYIEYGAAVLVLLVCVFGLAAHWMACIWYSIGDYEIFDEDTKTIRNNSWLYQLAMDIGTPY  430
Kv10.2  KCNH5  eag2  DHYLEYGAAVLVLLVCVFGLVAHWLACIWYSIGDYEVIDEVTNTIQIDSWLYQLALSIGTPY  400
Kv11.1  KCNH2  erg1  DRYSEYGAAVLFLLMCTFALIAHWLACIWYAIGNMEQPH---MD-SRIGWLHNLGDQIGKPY  597
Kv11.2  KCNH6  erg2  DRYSEYGAAVLFLLMCTFALIAHWLACIWYAIGNVERPY---LE-HKIGWLDSLGVQLGKRY  448
Kv11.3  KCNH7  erg3  DRYSEYGAAVLMLLMCIFALIAHWLACIWYAIGNVERPY---LT-DKIGWLDSLGQQIGKRY  599
Kv12.1  KCNH8  elk1  DRYSQHSTIVLTLLMSMFALLAHWMACIWYVIGKMEREDNSLLK-WEVGWLHELGKRLESPY  406
Kv12.2  KCNH3  elk2  DRYSQYSAVVLTLLMAVFALLAHWVACVWFYIGQREIESSESEL-PEIGWLQELARRLETPY  410
Kv12.3  KCNH4  elk3  ERYSQCSAVVLTLLMSVFALLAHWMACIWYVIGRREMEANDPLL-WDIGWLHELGKRLEVPY  412
                     ::* : .: ** **:. *.* ***:**:*: **  *            .** .*.  :   *
                                                                  P
                                                                             SF
Kv10.1  KCNH1  eag1  QFNGSG---------------------SG-KWEGGPSKNSVYISSLYFTMTSLTSVGFGNIA  470
Kv10.2  KCNH5  eag2  RYNTS----------------------AG-IWEGGPSKDSLYVSSLYFTMTSLTTIGFGNIA  439
Kv11.1  KCNH2  erg1  NSS---------------------------G-LGGPSIKDKYVTALYFTFSSLTSVGFGNVS  631
Kv11.2  KCNH6  erg2  NGS---------------------------DPASGPSVQDKYVTALYFTFSSLTSVGFGNVS  483
Kv11.3  KCNH7  erg3  NDS---------------------------DSSSGPSIKDKYVTALYFTFSSLTSVGFGNVS  634
Kv12.1  KCNH8  elk1  YGN---------------------------NTLGGPSIRSAYIAALYFTLSSLTSVGFGNVS  441
Kv12.2  KCNH3  elk2  YLVGRRPAGGNSSGQSDNCSSSSEANGTGLELLGGPSLRSAYITSLYFALSSLTSVGFGNVS  472
Kv12.3  KCNH4  elk3  V-N---------------------------GSVGGPSRRSAYIAALYFTLSSLTSVGFGNVC  446
                                                      .***  . *:::***:::***::****:.
                                            S6
Kv10.1  KCNH1  eag1  PSTDIEKIFAVAIMMIGSLLYATIFGNVTTIFQQMYANTNRYHEMLNSVRDFLKLYQVPKGL  532
Kv10.2  KCNH5  eag2  PTTDVEKMFSVAMMMVGSLLYATIFGNVTTIFQQMYANTNRYHEMLNNVRDFLKLYQVPKGL  501
Kv11.1  KCNH2  erg1  PNTNSEKIFSICVMLIGSLMYASIFGNVSAIIQRLYSGTARYHTQMLRVREFIRFHQIPNPL  693
Kv11.2  KCNH6  erg2  PNTNSEKVFSICVMLIGSLMYASIFGNVSAIIQRLYSGTARYHTQMLRVKEFIRFHQIPNPL  545
Kv11.3  KCNH7  erg3  PNTNSEKIFSICVMLIGSLMYASIFGNVSAIIQRLYSGTARYHMQMLRVKEFIRFHQIPNPL  696
Kv12.1  KCNH8  elk1  ANTDAEKIFSICTMLIGALMHALVFGNVTAIIQRMYSRWSLYHTRTKDLKDFIRVHHLPQQL  503
Kv12.2  KCNH3  elk2  ANTDTEKIFSICTMLIGALMHAVVFGNVTAIIQRMYARRFLYHSRTRDLRDYIRIHRIPKPL  534
Kv12.3  KCNH4  elk3  ANTDAEKIFSICTMLIGALMHAVVFGNVTAIIQRMYSRRSLYHSRMKDLKDFIRVHRLPRPL  508
                      .*: **:*::. *::*:*::* :****::*:*::*:    **     :::::::.:::*. *
```

B

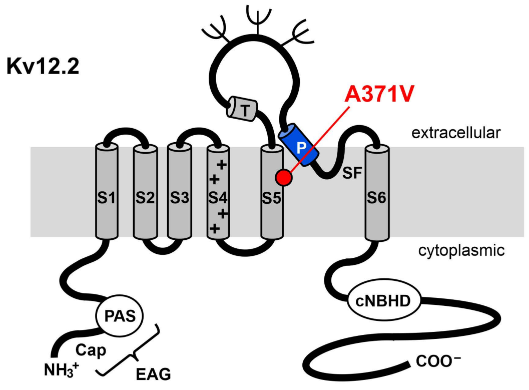

Supplementary Figure S1. Comparison of EAG channel protein sequences and schematic Kv12.2 α-subunit.
(A) UniProt single letter amino acid sequence alignment covering the transmembrane segment S5 (shaded grey), external pore loop with a turret helix (T, shaded grey), pore helix (P, shaded blue, white letters), potassium channel signature sequence involved in forming the selectivity filter (SF, bold letters), and transmembrane segment S6 (shaded grey), for all human EAG (Kv10 - Kv12) family members. Human genes and corresponding *Drosophila* orthologs are indicated; amino acid residue positions given on the right. Residues that are perfectly conserved (*****) or show strong (**:**) or weak (**.**) similarity are indicated. All Kv10-Kv12 subfamily members possess a conserved alanine residue (A, green) in S5 and the sequence GFGN in the SF. Note that Kv12.2 possesses an extended external pore loop harboring three N-glycosylation sites (bold letters). A phenylalanine residue (F) in the pore helix of Kv11 subfamily members, known to be relevant for ligand binding and inactivation gating in Kv11.1 (HERG; [54,55]), is replaced by a leucine residue (L) in Kv12 channels (magenta for Kv11.1 and Kv12.2).
(B) Location of the amino acid substitution in the Kv12.2 α-subunit. The channel protein contains six transmembrane segments (S1-S6) flanked by cytoplasmic amino (NH_3^+) and carboxy (COO^-) termini (extracellular and cytoplasmic side indicated). Positively charged amino acid residues in S4 constitute the voltage sensor, and a re-entrant pore loop between S5 and S6 harbors a turret helix (T), a pore helix (P), and the selectivity filter sequence (SF). Note the large extracellular S5/P linker domain with three N-glycosylation sites, typical for Kv12.2. The conserved N-terminal structure of EAG channels consists of a PAS (Per-Arnt-Sim) domain and a more distal PAS cap. A cyclic nucleotide-binding homology domain (cNBHD) is located in the cytoplasmic C-terminus. The amino acid substitution A371V in S5 is indicated by a red dot.

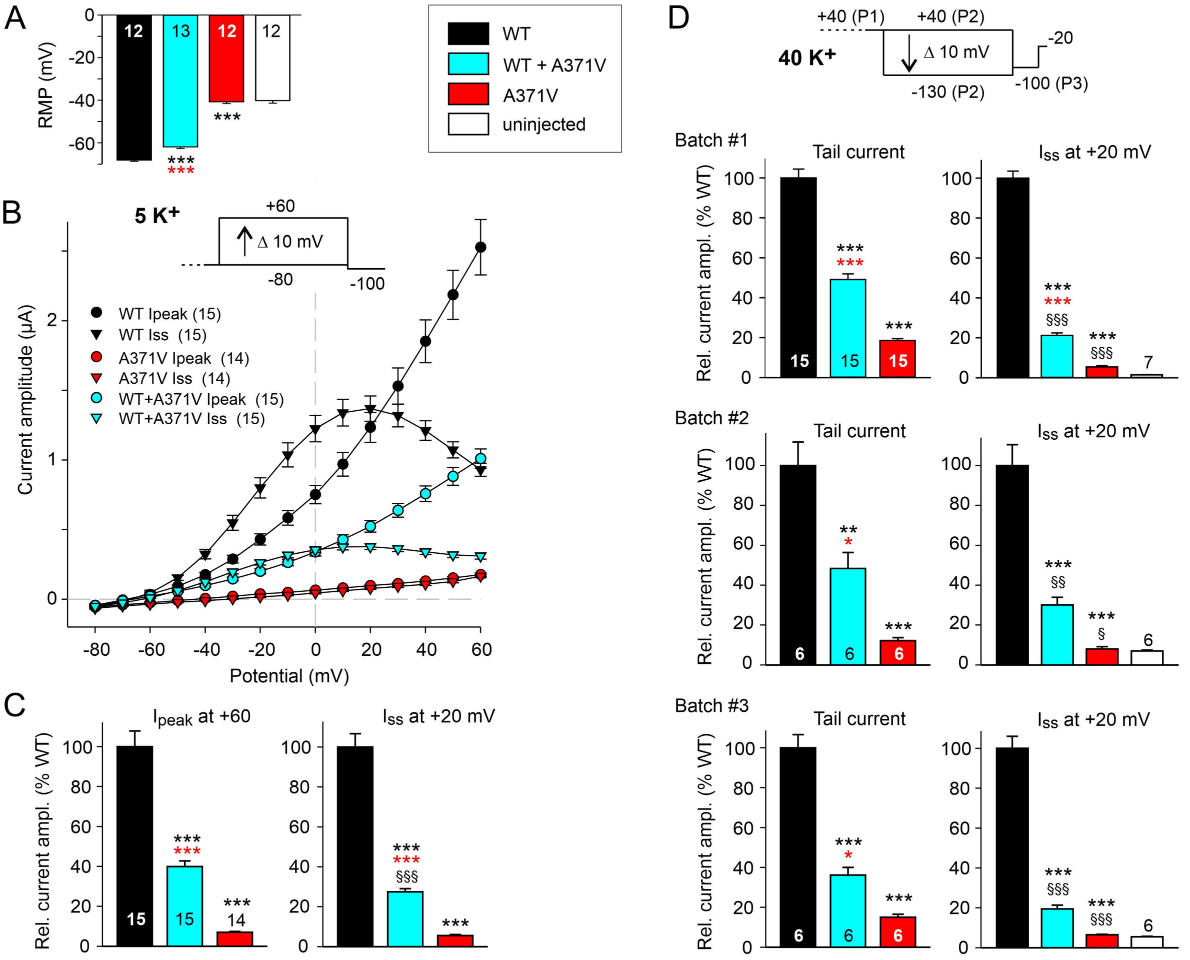

Supplementary Figure S2. Confirmation of effects of the Kv12.2 mutant A371V in 5 K^+ and analysis of experiments in 40 K^+ using oocytes from different *Xenopus laevis* donor frogs.

Comparative experiments were performed on oocytes injected with 5 ng WT (black), 5 ng A371V (red) or 2.5 ng of each WT + A371V Kv12.2 cRNA (cyan) as described previously [1]. Number of different oocytes used in parallel experiments is given in the bar plots. Data for uninjected oocytes from the same donor frog are given for information and have not been included in the statistical analysis. Black asterisks indicate a significant difference to WT, red asterisks indicate a significant difference to A371V data presented in the same diagram; *** $p<0.001$; ** $p<0.01$; * $p<0.05$; one-way ANOVA with *post hoc* Bonferroni's test for multiple pairwise comparison.

(A) Resting membrane potentials (RMP; means ± SEM) measured upon impalement of the voltage-electrode. Experiments performed in 5 K^+ external solution.

(B) and (C) Data analysis of two-electrode voltage clamp experiments performed in 5 K^+ external solution with the activation protocol (see inset in B). Analysis of experiments illustrated in *Figure 1A*. (B) Current-voltage (I-V) relationships for the peak currents (I_{peak}, circles) and the steady-state currents at the end of the test pulse (I_{ss}, triangles). Peak currents were determined as described previously [1].

(C) Significantly stronger relative suppression of the sustained current (I_{ss}) at +20 mV compared to the suppression of the peak current (I_{peak}) at +60 mV (§§§: $p<0.001$; paired t-test).

(D) Comparison of the effects of A371V on tail currents at -100 mV and on sustained currents at +20 mV. Data analysis of experiments performed in 40 K^+ external solution with the deactivation protocol (indicated on top) as shown in *Figure 1B-D* using oocytes from three different donor frogs. Left panels: means ± SEM of tail current amplitudes obtained from Boltzmann fits were normalized to the mean current amplitude of WT Kv12.2; results from analyses as shown in *Figure 1D*. Right panels: means ± SEM of sustained current amplitudes at +20 mV, normalized to the corresponding mean current amplitude of WT Kv12.2; data from results as shown in *Figure 1C*. Significantly stronger suppression of the sustained current (I_{ss}) at +20 mV compared to the suppression of the tail current at -100 mV is indicated by §§§ $p<0.001$, §§ $p<0.01$ or § $p<0.05$; paired t-test). Experiments illustrated in *Figure 1B-D* were performed in oocytes of batch #1. Identical experiments were performed in oocytes of two additional batches of oocytes (#2 and #3).

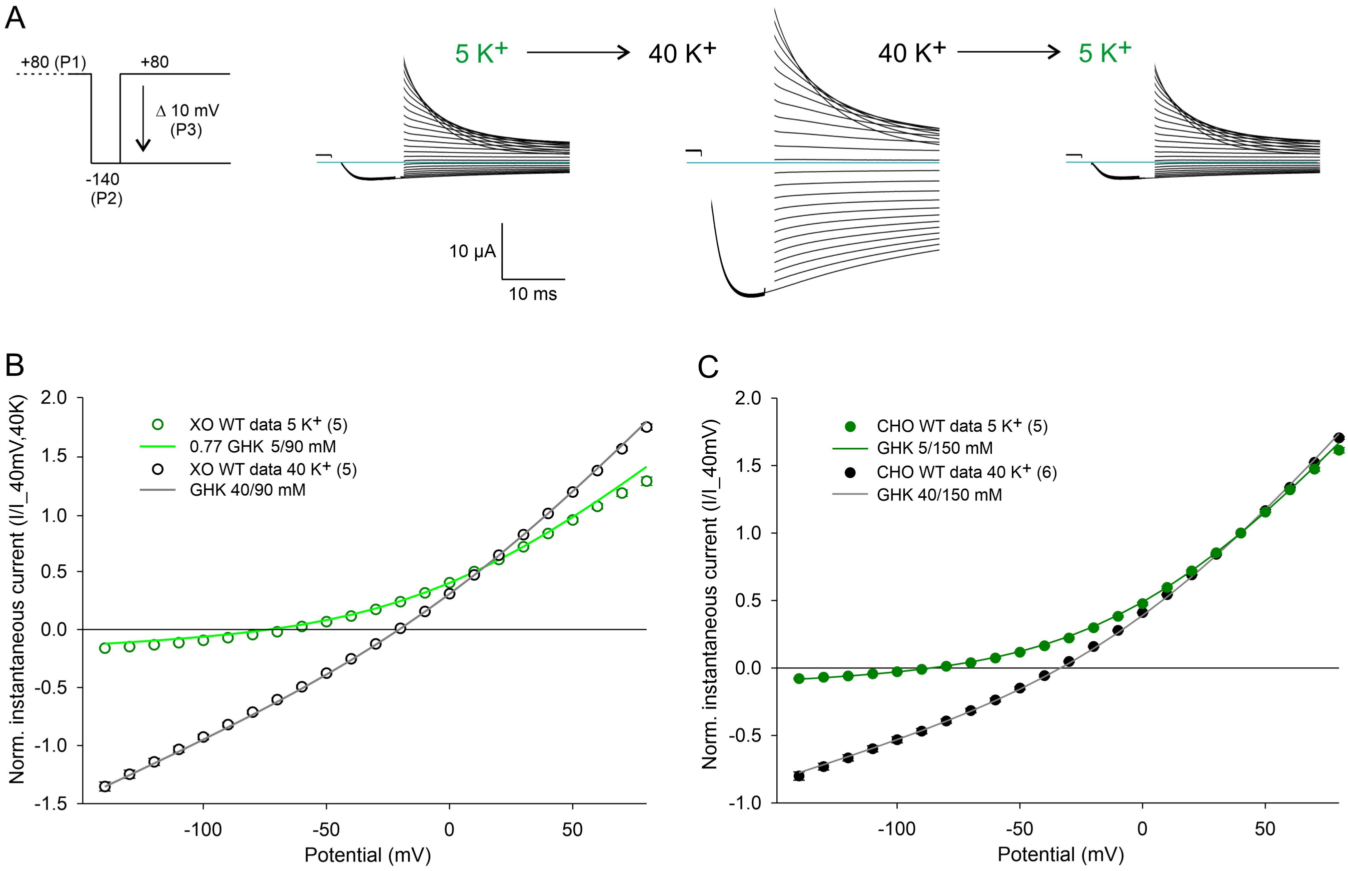

Supplementary Figure S3. Kv12.2 channels obey the Goldman-Hodgkin-Katz (GHK) current equation.
The voltage dependence of current passing through open Kv12.2 channels was described by the GHK current equation [18] to account for unequal K^+ concentrations in the external ($[K^+]_o$) and internal ($[K^+]_i$) solution:

$$I_K = P_K * E * \frac{F^2}{RT} * \frac{[K^+]_i - [K^+]_o \times exp\,(-\frac{E*F}{RT})}{1 - exp\,(-\frac{E*F}{RT})}$$

with E as the membrane potential, and P_K as K^+ permeability. P_K was adjusted to result in a normalized I_K value of 1 at +40 mV.

(A) Families of WT Kv12.2 current traces recorded in *Xenopus* oocytes (XO) with the indicated triple pulse protocol. From a holding potential of -20 mV (not shown), a 250 ms voltage step to +80 mV (P1) was applied to completely activate and inactivate the channels, followed by a short constant P2 pulse to -140 mV to open the channels by fast recovery from inactivation. Open channels were then subjected to variable P3 pulses ranging from +80 to -140 mV. The instantaneous current amplitude at the start of the P3 pulses served to assess the channel conductance in external solution with physiological (5 mM; 5 K^+) compared to elevated (40 mM; 40 K^+) K^+ concentration. Capacitive transients are blanked out for clarity. The oocyte was consecutively superfused with solution containing 5 mM, then 40 mM, and again 5 mM K^+.

(B) P3 instantaneous current amplitudes (means ± SEM) from experiments as shown in (A), as a function of the variable P3 potential. Current amplitudes were determined by fitting exponential functions to the P3 current decay following the capacitive current component and extrapolating the amplitude back to the start of the P3 pulse. Paired data were normalized to the current amplitude measured in 40 mM K^+ at +40 mV P3 potential. Continuous lines are similarly normalized results of the GHK current equation assuming an intracellular *Xenopus* oocyte K^+ concentration of 90 mM for calculation. Additionally, the GHK curve for 5 mM external K^+ was scaled down by a factor of 0.77 to better fit the experimental data.

(C) Kv12.2 WT currents were recorded from transfected CHO cells with the same triple pulse protocol as shown in (A) in external solution containing either 5 or 40 mM K^+ (unpaired experiments). The intracellular K^+ concentration was 150 mM. Instantaneous current amplitudes (means ± SEM) as a function of the P3 potential. Prior to averaging, data of single experiments were normalized to the extrapolated value of the instantaneous current amplitude at +40 mV. Continuous lines are similarly normalized results of the GHK current equation.

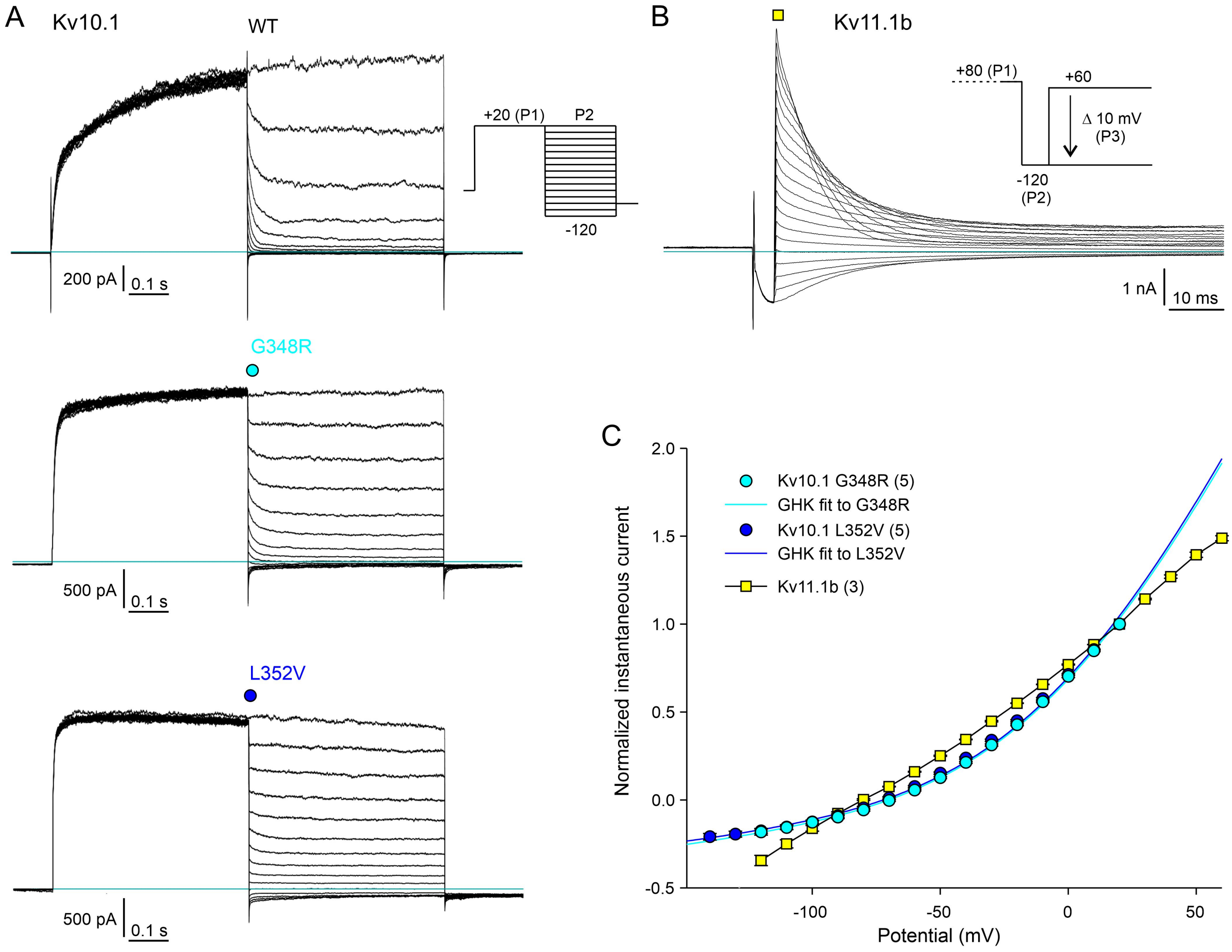

Supplementary Figure S4. Kv10.1, but not Kv11.1b channels obey the Goldman-Hodgkin-Katz (GHK) current equation.

(A) Families of current traces recorded in extracellular solution containing 5 mM K^+ from HEK293 cells transfected with cDNA encoding WT or mutant (G348R and L352V) Kv10.1 (heag1) channels, as described previously [44,61]. Compared to WT, the channel mutants deactivate much slower and the voltage dependence of channel deactivation is significantly shifted to more negative potentials, allowing reliable determination of instantaneous current amplitudes at the start of the P2 pulse (as indicated).

(B) Families of current traces recorded in external solution with 5 mM K^+ from CHO cells transfected with cDNA encoding Kv11.1b (erg1b) channels, as described previously [61]. Triple pulse protocol indicated.

(C) Instantaneous current amplitudes (means ± SEM) of P2 (Kv10.1) or P3 (Kv11.1b) as a function of the pulse potential. Prior to averaging, data of single experiments were normalized to the extrapolated value of the instantaneous current amplitude at +20 mV. Continuous lines (cyan and blue) denote fits of a simplified GHK current equation f =a*x*(1-exp(-(x-b)/25))/(1-exp(-x/25)) [39,44,45] to the mean data obtained for the Kv10.1 mutants G348R and L352V, respectively. Kv11.1b data could not be fitted with the GHK current equation and data points were connected by straight lines.

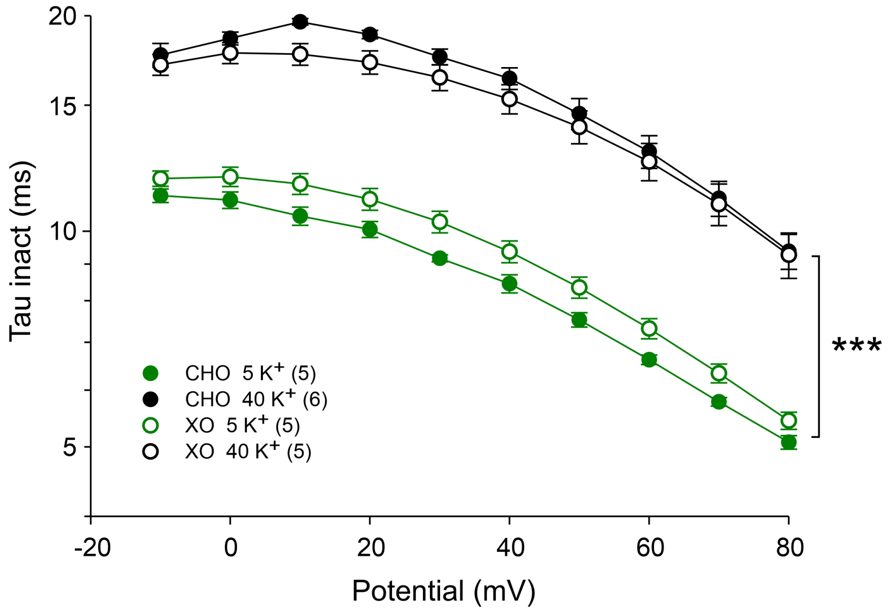

Supplementary Figure S5. High external K^+ slows Kv12.2 channel inactivation.
Time constants of WT Kv12.2 channel inactivation (means ± SEM) in 5 mM (5 K^+; green) and 40 mM (40 K^+; black) external K^+, determined from experiments as shown in *Figure S3A* by fitting a single exponential function to the P3 current decay after the capacitive current component. Experiments were performed in *Xenopus* oocytes (XO, paired data; open symbols) and in CHO cells (unpaired data; filled symbols); *** $p<0.001$ at all potentials; paired and unpaired t-test, respectively.

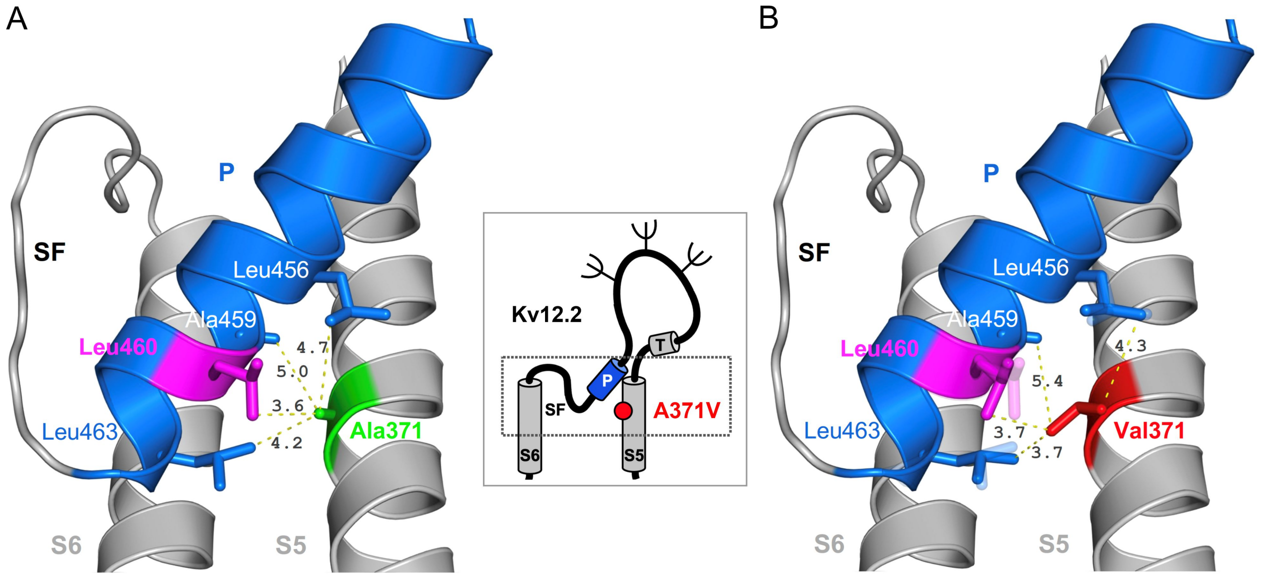

Supplementary Figure S6. Putative intramolecular interaction sites at position 371 in Kv12.2.
Structural homology modeling using UniProt (https://www.uniprot.org/), SWISS-MODEL (Center for Molecular Life Sciences, University of Basel; https://swissmodel.expasy.org/) and PyMOL (Schrödinger, New York, NY, USA) was performed based on the amino acid sequence of human Kv12.2 (UniProt: Q9ULD8) and the structural coordinates of Kv11.1 (PDB: 5VA2; [53]). (A) and (B) Enlarged views of the section indicated in the inset showing a membrane topology cartoon restricted to S5, extended external pore loop including T, N-glycosylation sites and P, SF and S6. Extracellular ends of the S5 and S6 transmembrane helices are shown for a wild-type (panel A; Ala371, green) and a mutant Kv12.2 α-subunit (panel B; Ala371 substituted with Val, red). The A371V amino acid substitution (indicated by a red dot in the inset) was computer-simulated with PyMOL considering the most likely backbone-dependent rotamer orientation (one out of three with a probability of 91.8%), and the putative steric consequences of the amino acid substitution within a distance of 5 Å was taken into account. The overlay in (B) illustrates the steric rearrangements in P proposed by this structural modeling approach, especially for Leu460 (magenta), which is homologous to a phenylalanine residue in Kv11.1 (F619; see alignment in *Figure S1B*). The shortest distances (in Å) between Ala371 (A) or Val371 (B) in S5 and the depicted residues in P were measured.

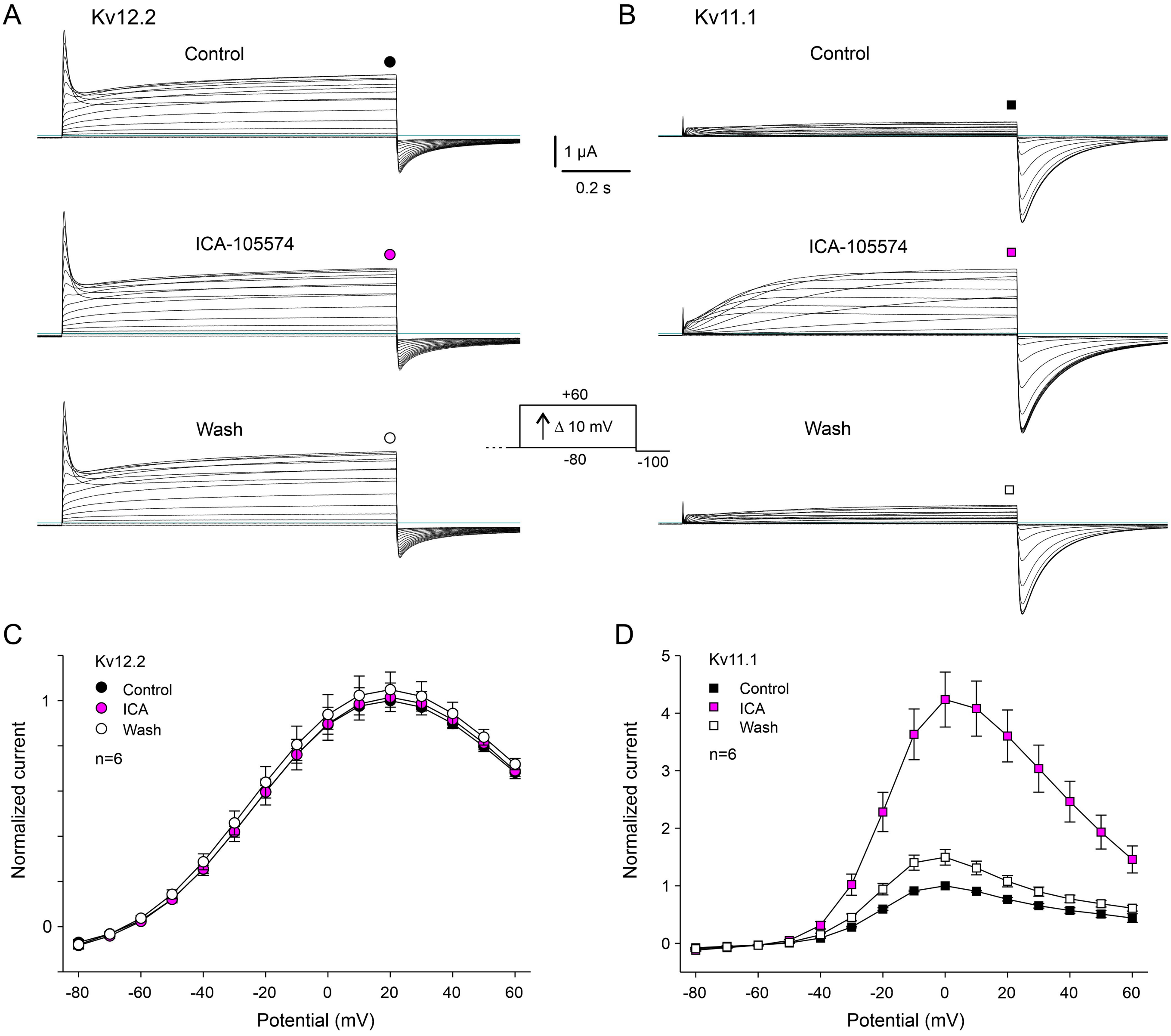

Supplementary Figure S7. HERG (Kv11.1) channel activator ICA-105574 does not affect Kv12.2.

(A) and (B) Comparative parallel experiments on Kv12.2 (A) and Kv11.1a (B) [61] were performed in *Xenopus* oocytes to investigate the effect of bath application of 20 µM ICA-105574. Examples of families of current traces recorded with the indicated pulse protocol in 5 K^+ external solution under control conditions (upper panels), at least 3 minutes after the start of ICA-105574 application (middle panels), and some minutes after the start of the wash (lower panels). To avoid data contamination by current run-up or run-down, the experimental protocol was kept short and no attempt was made to reach a steady-state of the ICA-105574 effect on Kv11.1. Drug solutions were prepared fresh from a 10 mM stock in DMSO for each set of parallel experiments on Kv12.2 and Kv11.1.

(C) and (D) Sustained current amplitude (means ± SEM); data were normalized to the control value obtained at +20 mV (C, Kv12.2) or at 0 mV (D, Kv11.1).

Supplementary Table S1. Voltage dependence and kinetics of WT and mutant Kv12.2 channel gating determined with 40 mM external K^+.

Related	Steady-state	XO	WT			A371V			Diff. to WT	WT + A371V (0.5+0.5)			Diff. to WT			
figure	parameter	Fit parameter	mean	SEM	n	mean	SEM	n	p	mean	SEM	n	p			
Figure 1D	Deactivation	V1/2 (mV)	-45.9	1.04	15	-35.17	1.02	15	<0.001	-48.89	0.81	15	0.065			
	(isochronal 1-s)	k (mV)	14.5	0.23	15	18.98	0.20	15	<0.001	16.37	0.14	15	<0.001			
		CHO cells	WT transfection			A371V transfection			Diff. to WT	WT + A371V (1+1) co-injection			Diff. to WT	WT injection		
		Fit parameter	mean	SEM	n	mean	SEM	n	p	mean	SEM	n	p	mean	SEM	n
Figure 2C	Deactivation	V1/2 (mV)	-65.4	1.30	14	-45.8	1.43	13	2.21E-10	-67.0	1.91	11	0.999	-67.1	2.31	8
	(isochronal 1-s)	k (mV)	18.2	0.37	14	21.3	0.60	13	1.18E-04	20.4	0.98	11	0.0361	17.0	1.06	8
Figure 3B	Activation	V1/2 (mV)	-54.3	1.55	14	-37.4	3.36	8	4.23E-05	-48.9	2.18	9	0.110	-54.3	1.91	6
		k (mV)	16.3	0.47	14	22.4	0.54	8	1.02E-07	19.8	0.42	9	0.00247	16.5	0.89	6
Figure 4C	Inactivation	V1/2 (mV)	-2.8	1.57	13	-120.4	1.46	6	2.36E-19	-42.8	4.89	10	9.32E-06	1.9	2.23	6
		k (mV)	39.2	0.84	13	33.0	1.69	6	0.00173	42.2	0.80	10	0.00435	37.8	1.03	6
		Test poten-	WT transfection			A371V transfection			Diff. to WT	WT + A371V (1+1) co-injection			Diff. to WT	WT injection		
	Channel kinetics	tial (mV)	mean	SEM	n	mean	SEM	n	p	mean	SEM	n	p	mean	SEM	n
Figure 2D	Tau fast, deact	-80	72.1	1.65	15	58.9	2.76	12	0.000232	69.2	3.79	10	0.149	79.1	5.65	8
	(ms)	-100	63.7	2.00	15	51.2	2.13	12	0.000254	62.0	3.21	10	0.0843	71.8	4.43	8
		-120	53.4	2.23	15	41.1	2.03	12	0.000526	51.2	2.75	10	0.012	64.6	4.00	8
Figure 2D	Tau slow, deact	-80	408	18	15	333	23	12	0.01450	462	88	10	0.975	459	58	8
	(ms)	-100	314	15	15	255	16	12	0.01140	288	15	10	0.076	389	57	8
		-120	249	14	15	190	14	12	0.00564	263	21	10	0.836	268	15	7
Figure 2D	Fraction fast	-80	0.556	0.019	15	0.602	0.028	12	0.176	0.494	0.026	10	0.856	0.501	0.027	8
	deactivation	-100	0.652	0.017	15	0.649	0.031	12	0.947	0.554	0.028	10	0.874	0.561	0.032	8
	f/(f+s)	-120	0.704	0.015	15	0.637	0.027	12	0.0281	0.593	0.042	10	0.461	0.632	0.027	8
Figure 2E	Tau recov	-80	6.46	0.19	15	3.05	0.24	11	3.67E-11	5.26	0.49	9	0.112	6.23	0.27	8
	(ms)	-100	4.54	0.17	15	1.90	0.12	11	1.65E-11	3.45	0.36	9	0.0788	4.27	0.21	8
		-120	3.09	0.12	15	1.10	0.07	11	1.42E-12	2.13	0.12	9	0.00136	2.80	0.13	8
Figure 3D	Tau fast, act (ms)	20	34.6	3.4	14	43.1	5.8	8	0.191	66.4	8.5	9	0.371	55.9	5.2	6
	Tau slow, act (ms)	20	593	68	14	732	69	8	0.198	1171	157	9	0.0228	638	89	6
	f/(f+s)	20	0.507	0.012	14	0.422	0.022	8	0.00167	0.447	0.021	9	0.0491	0.509	0.015	6
Figure 4D	Tau inact	80	10.15	0.26	13	2.00	0.08	6	1.41E-13	5.36	0.27	10	3.76E-08	9.83	0.35	7
	(ms)	60	13.96	0.51	13	2.55	0.07	6	2.94E-11	7.00	0.38	10	1.83E-08	12.82	0.35	7
		40	17.06	0.47	13	3.14	0.10	6	3.84E-13	8.70	0.48	10	2.79E-08	15.43	0.35	7
		20	18.02	0.27	13	3.69	0.11	6	2.73E-17	9.92	0.54	10	5.78E-08	16.99	0.36	7
		0	18.41	0.27	13	4.11	0.15	6	4.07E-17	10.34	0.52	10	1.14E-06	16.49	0.57	7
		-20	17.10	0.34	13	3.82	0.08	6	3.50E-15	9.28	0.67	10	2.46E-04	15.46	1.23	7

Analysis of WT and A371V Kv12.2 currents recorded in *Xenopus* oocytes (XO) and in CHO cells in external solution with elevated K^+ concentration. Co-expression experiments (WT + A371V) were performed in a non-additive (0.5+0.5; same amount of total cRNA) or additive way (1+1; double total cDNA concentration). *p* values originate from one-way ANOVA with *post hoc* Dunnett's test (XO data) or from Student´s two-tailed unpaired t test (CHO data). Red *p* values indicate significant differences compared to WT data, green *p* values are >0.05.
